# An efficient and versatile synthesis of GlcNAcstatins—potent and selective *O*-GlcNAcase inhibitors built on the tetrahydroimidazo[1,2-*a*]pyridine scaffold

**DOI:** 10.1016/j.tet.2010.07.037

**Published:** 2010-09-25

**Authors:** Vladimir S. Borodkin, Daan M.F. van Aalten

**Affiliations:** Division of Molecular Microbiology, College of Life Sciences, University of Dundee, Dundee DD1 5EH, Scotland, UK

## Abstract

We report a novel approach to the synthesis of GlcNAcstatins—members of an emerging family of potent and selective inhibitors of peptidyl *O*-GlcNAc hydrolase build upon tetrahydroimidazo[1,2-*a*]pyridine scaffold. Making use of a streamlined synthetic sequence featuring de novo synthesis of imidazoles from glyoxal, ammonia and aldehydes, a properly functionalised linear GlcNAcstatin precursor has been efficiently prepared starting from methyl 3,4-*O*-(2′,3′-dimethoxybutane-2′,3′-diyl)-α-d-mannopyranoside. Subsequent ring closure of the linear precursor in an intramolecular S_N_2 process furnished the key fused d-mannose-imidazole GlcNAcstatin precursor in excellent yield. Finally, a sequence of transformations of this key intermediate granted expeditious access to a variety of the target compounds bearing a C(2)-phenethyl group and a range of *N*(8) acyl substituents. The versatility of the new approach stems from an appropriate choice of a set of acid labile permanent protecting groups on the monosaccharide starting material. Application was demonstrated by the synthesis of GlcNAcstatins containing polyunsaturated and thiol-containing amido substituents.

## Graphical abstract

**Figure:**
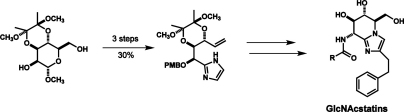

## 1 Introduction

Reversible posttranslational modification of cytoplasmic and nuclear proteins in eukaryotic cells by glycosylation of (surface exposed) serines and threonines with 2-acetamido-2-deoxy-β-d-glucopyranosyl residues (*O*-GlcNAc) is believed to play important roles in diverse cellular processes, such as transcription, translation, signal transduction and protein trafficking and degradation.^1–3^ There is evidence to suggest that the faulty interplay between O-GlcNAcylation and competitive or synergistic phosphorylation of specific proteins is involved in progression and pathology of several metabolic and neurodegenerative diseases. Thus an increased level of O-GlcNAcylation on proteins along the insulin signalling pathway has been proposed to be involved in insulin sensitivity,^4,5^ and hyper-phosphorylation of the microtubule-associated protein tau that marks the development of the Alzheimer disease was shown to be reciprocal with the abnormally low O-GlcNAcylation.^6^

The molecular mechanisms and biological implications of *O*-GlcNAc attachment/hydrolysis are currently the subject of intense multidisciplinary investigation. In a striking contrast to the conceptually related reversible protein phosphorylation, regulated by a plethora of highly specific kinases and phosphatases, the dynamic cycling of *O*-GlcNAc in eukaryotes is achieved by a single pair of enzymes: peptidyl *O*-GlcNAc transferase (OGT, CAZY family GT41^7^) and peptidyl *O*-GlcNAc hydrolase (OGA, GH84).

It has so far not been possible to study the cell biological effect of hypo-O-GlcNAcylation due to a lack of OGT inhibitors. However, OGA, with a number of stable functional substrate analogues available, provides an excellent opportunity to study the cellular effects of inhibitor-induced hyper-O-GlcNAcylation.

**Figure 1:**
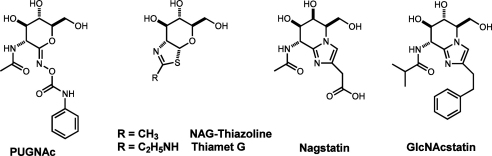


A number of potent OGA inhibitors have been developed in the recent decades, including PUGNAc (*K*_i_=50 nM, hOGA)^8–10^ and NAG-thiazoline (*K*_i_=70 nM, hOGA)^11,12^ (Fig. 1). Although both compounds are prone to hydrolysis in aqueous solutions and also potently inhibit lysosomal β-hexosaminidases (HexA/HexB), they became the standard tools for studying protein over O-GlcNAcylation. Recently, systematic variation of the size and electronic properties of the thiazoline substituent resulted in the discovery of the highly potent, selective and hydrolytically stable inhibitor Thiamet-G (*K*_i_=21±3 nM, hOGA).^13^

In parallel, a search for novel, potent and selective OGA inhibitors focussed our attention on the naturally occurring hexosaminidase inhibitor nagstatin^14,15^ (Fig. 1). Its unique tetrahydroimidazo[1,2-*a*]pyridine (fused sugar-imidazole) bicyclic scaffold and outstanding potency fuelled the research of Tatsuta et al. on the total synthesis of the parent compound and analogues.^16,17^ Ensuing work of Vasella et al. has established sugar-imidazoles among the most powerful β-glycosidase inhibitors.^18^ The origin of this potency was attributed to the half-chair conformation of the bicyclic core mimicking the flattened geometry of the putative pyranosyl oxocarbenium ion during the hydrolysis of glycosidic bond. The importance of the [1,2-*a*] fused imidazole ring was deduced to stem from in-plane protonation of the pseudo-anomeric nitrogen atom with a laterally positioned catalytic acid, that would proceed along the same trajectory as protonation of a glycosidic bond to yield the β-anomeric oxygen leaving group.^19^ Additionally, in contradiction with the earlier Tatsuta’s data, C(2) substituted fused sugar-imidazoles were found to show improved inhibition over unsubstituted analogues. The effect, particularly profound in the phenethyl-substituted fused hexose-imidazoles,^20^ has been also replicated for the series of C(2) phenethyl-substituted fused pentose-imidazoles.^21^ Surprisingly, the inhibition of hexosaminidases with nagstatin and analogous fused 2-acetamido-2-deoxy sugar-imidazoles, was only tested very recently, when synthesis of *gluco*-nagstatin was reported by Vasella et al.^22,23^

Based on these data and the availability of the crystal structure of a bacterial OGA (bOGA) homologue from *Clostridium perfringens* in complex with PUGNAc^24^ we designed GlcNAcstatin **6** (Fig. 1)—the founding member of a novel family of OGA inhibitors built on the fused d-glucosamine-imidazole scaffold. In a preliminary communication we reported synthesis and enzymatic profiling of this compound to reveal its outstanding potency and selectivity (*K*_i_=4 pM, bOGA).^25^ Recently we reported the evaluation of a small set of GlcNAcstatins as inhibitors of human OGA to confirm that both potency and selectivity could also be achieved with the human enzyme.^26^

**Figure 2:**
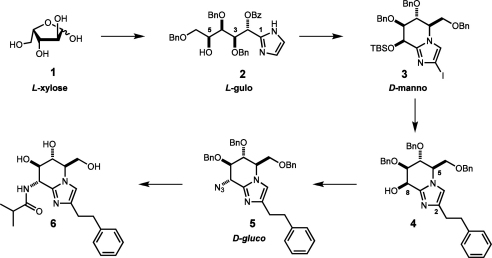


The synthesis of GlcNAcstatin was provisionally designed as a consolidation of two existing pathways to the fused sugar-imidazoles (Fig. 2).^25^ It exploited the highly efficient S_N_2 substitution of the OH(8) group in the *manno*-configured C(2)-branched derivative **4** (tetrahydroimidazo[1,2-*a*]pyridine numbering) leading to the *gluco*-azide **5** in accordance with Vasella’s findings.^23^ In turn, the key iodo-imidazole **3** was prepared using an extensively modified Tatsuta’s approach from the l-xylose **1** through the linear polyhydroxylated l-*gulo*-imidazole **2**.^16^ The overall competence of the synthesis, however, was undermined by the poor efficacy of the opening step, which required separation of mixtures of diastereomers and unproductive protective group exchange. Further progress on the synthesis of GlcNAcstatins aimed at the development of more potent/selective inhibitors of hOGA was hampered by routine supply of the required intermediates. Aiming at the synthesis of derivatives bearing unsaturated or thiol-containing amide substituents, the abolishment of an array of permanent benzyl protecting groups on the sugar moiety would result in optimization of the synthetic scheme. To fulfil these objectives we developed a novel synthetic approach to GlcNAcstatins, making use of de novo synthesis of linear polyhydroxylated imidazoles^21,27^ from glyoxal, ammonia and chiral aldehydes available by reductive cleavage of sugar primary iodides (Bernet–Vasella reaction).^28^

## 2 Results and discussion

**Scheme 1:**
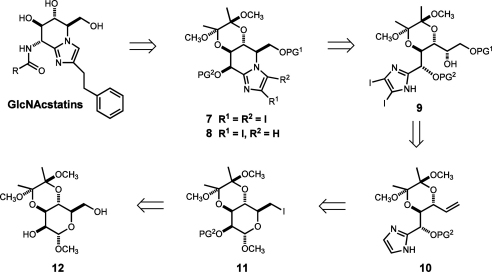


We identified methyl 3,4-*O*-(2′,3′-dimethoxybutane-2′,3′-diyl)-α-d-mannopyranoside **12**, easily available on a large scale,^29^ as the optimal monosaccharide starting material (Scheme 1). Robust 3,4-*O*-bis-acetal protecting group in **12**, meeting the criteria for the acid labile permanent protecting group, would keep corresponding hydroxyls intact through the synthesis. The rigid bicyclic framework of **12** would also allow efficient differentiation of the remaining hydroxyl groups via selective alkylation of the OH(2) according to the Ley’s protocol^30^ thus securing a streamlined subsequent introduction of the 6-iodo substituent (**12**→**11**). Reductive cleavage of **11** would then afford the open-chain aldehyde, which is instantly ready for the three component construction of the imidazole ring (**11**→**10**). Asymmetric dihydroxylation of the double bond in **10** followed by selective protection of OH(6) with suitable silyl ether (acid labile) and bis-iodination of the imidazole ring would generate the required l-gulo configured cyclisation precursor **9**.

We anticipated that after this point a synthetic sequence comprising of tetrahydroimidazo[1,2-*a*]pyridine ring closure (**9**→**7**) and selective C(3) deiodination of the imidazole (**7**→**8**) would give access to the key fused d-mannose-imidazole precursor of GlcNAcstatins **8**, similar to the one successfully transformed into GlcNAcstatin in the provisional synthesis.^25^

**Scheme 2:**
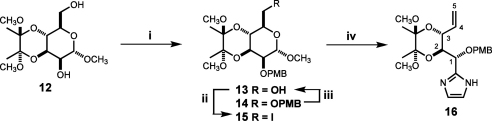
Reagents and conditions: (i) PMBCl, NaH, *n*-Bu_4_NI, DMF, rt, 16 h, 50%; (ii) I_2_, ImH, PPh_3_, toluene, 70 °C, 3 h, 75%; (iii) H_2_, Pd/C, MeOH/EtOAc, 3 h, 50%; (iv) (a) Zn, THF/H_2_O (10:1), 65 °C, 1 h, (b) 40% aqueous glyoxal, 7 M NH_3_/MeOH, 0–70 °C, 2 h, 70%.

In the event selective *p*-methoxybenzylation of the starting mannoside **12** with 1.15 equiv of *p*-methoxybenzyl chloride (PMBCl) in the presence of *n*-Bu_4_NI provided the expected 2-*O*-PMB ether **13** in about 50% yield along with 25% of 2,6-di-*O*-PMB derivative **14** (Scheme 2). Notably, the control experiment with benzyl bromide afforded the corresponding 2-*O*-Bn ether in 78% yield, in agreement with the original data.^30^ We found that the primary PMB group in compound **14** could be selectively removed by hydrogenolysis under controlled conditions to give a 10:1:9 mixture of 2-*O*- and 6-*O*-monoalkylated derivatives and the starting material **14** from which the 2-*O*-PMB ether **13** was isolated in 50% yield after flash chromatography. This observation was used advantageously to increase the overall yield of the requisite compound **13** to 60%, starting from the bis-acetal **12**. Substitution of the OH(6) in **13** was then achieved using PPh_3_/imidazole/I_2_ in hot toluene^31^ to furnish the iodo-derivative **15** in 75% yield on 30 mmol scale. The ensuing reductive splitting of the pyranoside ring of **15** using a variant of the Bernet–Vasella reaction^28^ with freshly activated zinc in aqueous THF at 65 °C gave the intermediate open-chain aldehyde, which on treatment with 40% glyoxal solution in 7 M methanolic ammonia initially at 0 °C and then at 70 °C for 2 h consistently produced the key intermediate imidazole **16** in a rewarding 70% overall yield.

**Scheme 3:**
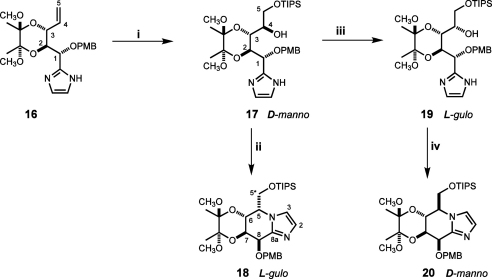
Reagents and conditions: (i) (a) K_2_(OsO_4_)/K_3_[Fe(CN)_6_], K_2_CO_3_, CH_3_SO_2_NH_2_, *t*-BuOH/THF/water, rt, 24 h, (b) TIPSCl, Py, 50 °C, 16 h, 78%; (ii) Tf_2_O, pyridine, −15 °C tort, 1 h, 92%; (iii) (a) (COCl)_2_, DMSO, DCM, −60 °C then Et_3_N, (b) NaBH_4_, EtOH, rt, 16 h, 80%; (iv) Tf_2_O, Py, C_2_H_4_Cl_2_, −15 °C then 50 °C, 3 h, 90%.

Initial catalytic dihydroxylation of the double bond in the open-chain imidazole **16** with K_2_(OsO_4_)/K_3_[Fe(CN)_6_] was found to be highly stereoselective, yet unfortunately providing the unwanted d-*manno*-configured derivative **17** as a sole product isolated in 78% yield after selective silylation of the primary hydroxy group with triisopropyl chlorosilane (Scheme 3). Strikingly, running the reaction in the presence of varying amounts of the β-selective (DHQD)_2_-PHAL ligand (0.1–5%) did not affect the stereochemical outcome or efficiency of the process, suggesting an overpowering intrinsic facial stereospecificity of the substrate.^32^ The absolute configuration of **17** was established after its transformation into the fused sugar-imidazole derivative **18** by treatment with Tf_2_O in pyridine for 1 h at rt. Formation of the bicyclic compound was confirmed by HRMS and NMR spectra. In particular, the presence of the (C8a)–(H5) and (C3)–(H5) (tetrahydroimidazo[1,2-*a*]pyridine numbering) cross-peaks in the HMBC spectra proved that imidazole moiety was covalently bound to C(5). Assuming clean inversion of the configuration at C(5) occurred during cyclisation, observation of the nuclear Overhauser effect (NOE) between protons H(7) and H(5^∗^a, b) in compound **18** was only compatible with its l-*gulo* configuration and, respectively, d-*manno* configuration of the starting compound **17**.

The origin of the exceptional substrate-controlled selectivity of the dihydroxylation reaction is not immediately obvious. It is possible that it could stem from formation of a complex of osmium tetroxide with the imidazole; in this case, formation of the d-*manno*-configured product would result from the intramolecular delivery of the reagent onto the eclipsed rotamer of the double bond. On the other hand, the same stereochemical outcome could result from an effective shielding of one of the diastereotopic faces of the double bond by the bulky C1 substituent. In this case, however, the double bond should react in a less favourable bisecting conformation. Whatever the origin of the unique stereoselectivity of the process, the reported observations provide a rare glimpse on the barely explored stereodirecting properties of bis-acetal protected substrates in dihydroxylation reactions.^33^

In an attempt to invert the configuration of OH(4) in compound **17** we have found that it remained unchanged in the Mitsunobu esterification with DIAD/PPh_3_/*p*-nitrobenzoic acid. However, a simple two-step Swern oxidation^34^—NaBH_4_ reduction sequence cleanly provided a 1:10 mixture of the epimeric alcohols **17** and **19**. The ample difference of the retention factors allowed the individual isomers to be isolated in 9% and 80% yields, respectively, by chromatographic separation. Operational simplicity and scalability of the devised sequence resulted in only a limited exploration of alternative, more selective, reducing agents. Addition of cerium chloride (NaBH_4_, CeCl_3_·7H_2_O/MeOH; −78 °C to rt) did not change the stereoselectivity, whereas DIBAL failed to reduce the intermediate ketone even at ambient temperature.

The configuration of OH(4) in compound **19** was formally established after closure of the bicyclic compound **20**, which required heating of the intermediate bis-triflate at 50 °C for 3 h to go to completion (Scheme 3). The observation of the NOE between H(6) and H(5^∗^a, b) in compound **20** established its d-*manno* absolute configuration and consequently the l-*gulo* configuration of compound **19**.

Similar to the stereochemical outcome of the dihydroxylation of **16**, the formal Felkin–Anh stereochemistry of the reduction of the intermediate ketone leading to the preferential formation of the alcohol **19** is difficult to rationalise. Due to the presence of the rigid bis-acetal framework adorned with the imidazole ring and multiple alkoxy substituents, a choice of opportunities for remote chelate formation is endless making the analysis in terms of chelate or non-chelate models not conclusive.

**Scheme 4:**
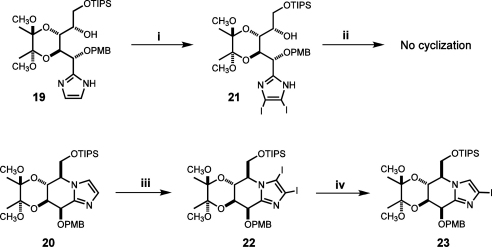
Reagents and conditions: (i) NIS 2.5 equiv, MeCN, 6 h, rt, 95%; (ii) Tf_2_O, Py, C_2_H_4_Cl_2_, −15 °C then 50 °C; (iii) NIS 10 equiv, DMF, 85 °C, 36 h, 80% or NIS 3 equiv, MeCN, PPTS, 80 °C, 3 h, 65%; (iv) EtMgBr, THF, 0 °C, 10 min, 88%.

In line with our previous findings^25^ we initially decided to convert the open-chain imidazole **19** into the bis-iodo-derivative **21** prior to cyclisation to avoid the drastic iodination conditions used by Vasella et al. for iodination of preformed fused sugar-imidazoles.^35^ As expected, iodination of **19** with 2.5 equiv of NIS in MeCN proceeded smoothly to give the requisite compound **21** (Scheme 4). However, all attempts to effect its annulation into the targeted tetrahydroimidazo[l,2-*a*]pyridine derivative **22** failed in marked contrast with the highly effective cyclisation of the parent non-iodinated compound **19** and previously observed smooth cyclisation of the benzylated/benzoylated analogue not possessing bis-acetal protection.^25^ The lack of reactivity may be explained by the spatial congestion developing in a transition state between the iodine substituent at C(3) position of the imidazole ring and the bulky primary triisopropylsilyloxy group.

Finally we found that post-cyclisation iodination of the fused sugar-imidazole **20** using an even more vigorous variant of Vasella’s original conditions (10 equiv NIS, DMF, 85 °C, 36 h) furnished the key diiodide **22** as slightly yellow oil in 80% yield after chromatographic purification of the very dark reaction mixture (Scheme 4). Alternatively, iodination of **20** could be performed using 3 equiv of NIS in MeCN at 80 °C in the presence of 1 equiv of PPTS^36^ giving the requisite compound **22** in 60% yield. The detected formation of *p*-methoxybenzyl iodide as a by-product pointed at an inadequate stability of the PMB protective group towards the NIS/PPTS combination as a principal reason for the diminished efficacy of bis-iodination. In the concluding step, the regioselective mono-deiodination of **22** at C(3) with EtMgBr/THF at 0 °C was executed without incident to give the key mono-iodoimidazole **23** in 88% yield.

**Scheme 5:**
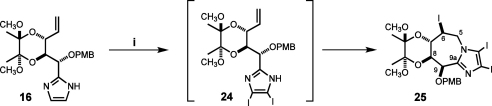
Reagents and conditions: (i) NIS 3 equiv, MeCN, 6 h, rt, 78%.

It is worth noting that explorative one-step iodination/iodocyclisation of the unsaturated open-chain imidazole **16** with 3 equiv of NIS resulted in transient formation of the bis-iodinated imidazole **24** and, finally, triply iodinated tetrahydro-5*H*-imidazo[1,2-*a*]azepine derivative **25** in 78% yield as a result of apparent *anti*-Baldwin 6-*endo*-*trig* cyclisation (Scheme 5). The structure of the compound **25** was initially deduced from the high field shift of C(6) (*δ* 26.7) (imidazo-azepine numbering) in the ^13^C NMR spectrum, pointing at the iodine substitution site. This was further confirmed by observation of the (C9a)–(H5a)/(5b) cross-peaks in the HMBC spectrum, revealing covalent connection of the imidazole and the methylene group of the azepine ring. The configuration of the C(6) iodo substituent was established from the presence of the H(6)–H(8) cross-peak in the NOESY spectrum showing spatial proximity of the corresponding protons.

**Scheme 6:**
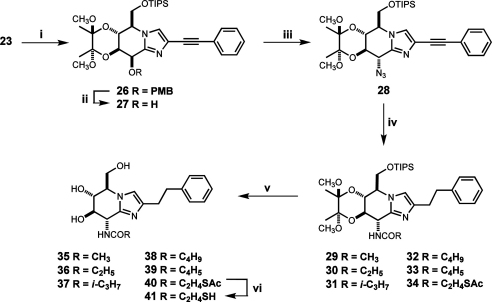
Reagents and conditions: (i) PhCCH, CuI, Et_3_N, Pd(PPh_3_)_4_, DMF, 80 °C, 16 h, 93%; (ii) DDQ, DCM/H_2_O, 4 h, 83%; (iii) DPPA, DBU, toluene, rt then 80 °C, 2 h, 93%; (iv) (a) H_2_, Pd/C, MeOH or EtOAc, 1 h (b) (RCO)_2_O, Et_3_N, DCM for **29**–**31** or PyBOP, DIPEA, RCO_2_H, DCM, rt, 16 h, for **32**–**34**; (v) TFA/H_2_O (95:5), rt, 36 h, 60–80%; (vi) DMF, MeONa/MeOH, DTT, 2 h, 56%.

Subsequently, a highly efficient Sonogashira coupling^37^ of the key iodoimidazole **23** with phenylacetylene provided the fully assembled precursor of GlcNAcstatins **26** with the C(2) phenylethynyl substituent in place in 93% yield (Scheme 6). Critical stereoselective conversion of the *manno*-configured **26** into the *gluco*-azide **28** was achieved in two steps starting with oxidative removal of the PMB protective group with DDQ (**26**→**27**; 83%) followed by azidation of the alcohol **27** with DPPA/DBU under optimized conditions (toluene, 80 °C, 2 h) to produce the requisite azide **28** in 93% yield as the single product. The *gluco*-configuration of the compound **28** was unambiguously deduced from the value of the corresponding coupling constant (*J*_7,8_ 9.5 Hz) as well as the presence of H(6)–H(8) cross-peak in the NOESY spectrum.

Starting from the azide **28** a panel of fully protected *N*-acylated GlcNAcstatins precursors **29**–**34** encompassed derivatives of acetic, propionic, *iso*-butyric, valeric, 2,4-pentanedioic^38^ and 3-(acetylthio)propionic acids has been prepared using a uniform sequence consisted of simultaneous hydrogenation both of the triple bond and the azido group over Pd catalyst followed by reaction of the intermediate amine with suitable acylating agents.

As the concluding step of the synthesis, removal of the permanent protecting groups (TIPS and 3,4-*O*-dimethoxybutanediyl bis-acetal) was intended to be achieved with acid hydrolysis in one step. In the event the reaction was found to be prohibitively slow. When solutions of a substrate in DCM were exposed to the increasing (10–50%) concentrations of TFA/water (95:5) in DCM, both protecting groups remained intact for a period of several hours. Moreover, it appeared that the TIPS group could be selectively removed by treatment with 4 M HCl in THF for 16 h, leaving the butanediyl bis-acetal protection mostly unchanged. Our findings appear to be in agreement with a singular report suggesting that removal of 3,4-*O*-bis-acetal protection in *N*-acetyl glucosamine derivatives could be a slow process.^39^ It seems that protonation of the imidazole ring of the fused *N*-acetylated glucosamine-imidazoles **29**–**34** contributes significantly to the acid stability of the butanediyl bis-acetal protection making it even more resistant towards hydrolysis than in *N*-acetyl glucosamine derivatives. Finally, global deprotection of **29**–**34** was achieved by 36 h treatment with aqueous TFA (95:5) to furnish the requisite compounds **35**–**40** in acceptable yields after reversed phase chromatography purification. To obtain the 3-thiopropionic acid derivative **41**, *S*-acetyl protection was finally removed from **40** with MeONa/MeOH in the presence of dithioerythrol.

## 3 Conclusions

We reported here a novel approach to the synthesis of a family of potent and selective inhibitors of peptidyl *O*-GlcNAcase (GlcNAcstatins) built on the fused d-glucosamine-imidazole scaffold. At the outset of the synthesis a short practical route to the densely functionalised open-chain imidazole bearing a terminal double bond has been developed using a novel combination of Bernet–Vasella reaction and de novo imidazole synthesis. Dihydroxylation of the double bond in this intermediate was found to be exclusive substrate controlled process leading to formation of the unwanted d-*manno*-configured product. The inversion of configuration of the OH(4) group has been achieved by a serendipitously found by-pass method using stereoselective reduction of the intermediate ketone. The obtained l-*gulo* substrate resisted all attempts to be cyclised into bicyclic derivative when iodine atoms have been preinstalled at the imidazole ring. In contrast, seamless cyclisation of the non-iodinated precursor into the requisite fused d-mannose-imidazole was easily achieved using the standard protocol. Ultimately, a Sonogashira reaction of C(2) iodo-derivative of the cyclised compound with phenylacetylene provided highly efficient access to the advanced intermediate in the GlcNAcstatin synthesis bearing a phenylethynyl substituent at the C(2) position. Starting from this compound, a set of GlcNAcstatins with assorted amido substituents has been prepared. Although the very last step of the synthesis, i.e., acidolytic removal of the permanent triisopropylsilyl and cyclic bis-acetal protections was found to be rather sluggish, the versatility of the new synthetic approach was clearly demonstrated by the preparation of GlcNAcstatin derivatives of 2,4-pentanedioic and 3-mercaptopropionic acids intrinsically incompatible with the hydrogenolytic deprotection routines. The new approach also gives access to rapid future exploration of alternative C(2) substituents that may yield even more potent, selective and cell-permeable GlcNAcstatin derivatives.

## 4 Experimental

### 4.1 General

All reactions were performed in oven-dried glassware under an inert atmosphere (argon) unless noted otherwise. Dichloromethane, acetonitrile, triethylamine were anhydrous grade solvents from Fluka kept over molecular sieves. Analytical thin layer chromatography (TLC) was performed on Merck silica gel 60 F_254_ aluminium plates (0.25 mm). Compounds were visualized by UV light, or by dipping the plate into acidic potassium permanganate aqueous solution followed by washing out the excess of the reagent, or by charring the plate at ca. 300 °C after dipping in a 5% phosphomolybdic acid solution in ethanol. Flash chromatographic separations were performed on Isco RediSep flash columns using Buchi gradient pump system. NMR spectra were recorded on Bruker AVANCE II 500 spectrometer. Splitting patterns of spectral multiplets are indicated as s, singlet; d, doublet; br s broad singlet; br d broad doublet; t triplet; quint quintet. Signals were assigned by means of DEPT, COSY, HSQC, and HMBC spectra. High-resolution mass spectra (HRMS) were obtained on a microTOF Bruker Daltonics instrument. Optical rotations were measured in chloroform on a Perkin–Elmer 343 polarimeter at 20 °C.

FT-IR spectra were recorded on Perkin–Elmer Spectrum BX instrument.

#### 4.1.1 (2′*S*,3′*S*)-Methyl 2-*O*-(4-methoxybenzyl)-3,4-*O*-(2′,3′-dimethoxybutane-2′,3′-diyl)-α-d-mannopyranoside (**13**) and (2′*S*,3′*S*)-methyl 2,6-di-*O*-(4-methoxybenzyl)-3,4-*O*-(2′,3′-dimethoxybutane-2′,3′-diyl)-α-d-mannopyranoside (**14**)

To a cooled (ice-bath) solution of **12** (15.16 g, 49.16 mmol) and *p*-methoxybenzyl chloride (7.5 mL, 55.31 mmol) in DMF (250 mL) sodium hydride (60% in oil; 4 g, 100 mmol) was added in portions. The reaction was stirred for 1 h and then removed from the ice-bath; *n*-Bu_4_NI (1.8 g, 4.87 mmol) was added at this moment. The reaction was further stirred for 16 h at rt. An excess of sodium hydride was quenched by careful addition of ice chips with external cooling (ice-bath). After gas evolution ceased the reaction was partitioned between water and ethyl acetate and the layers were separated. The organic layer was washed with water. The aqueous layers were additionally extracted with ethyl acetate. The combined organic layer was dried and concentrated. The residue was purified by flash chromatography in Tol/EA 5→50% to give and 11.37 g (26.5 mmol, 54%) of the title compound **13** as foam and 6.58 g (11.99 mmol, 25%) of the title compound **14** as clear syrup.

#### 4.1.2 Compound **13**

*δ*_H_ (500 MHz, CDCl_3_) 7.38–7.32 (2H, m, CH_2_*Ph*OMe), 6.88–6.82 (2H, m, CH_2_*Ph*OMe), 4.87 and 4.58 (AB spectrum, *J* 11.4 Hz, C*H*_2_PhOMe), 4.63 (1H, d, *J*_1,2_ 1.3 Hz, H-1), 4.18 (1H, t, *J*_3,4_=*J*_4,5_=10.3 Hz, H-4), 4.05 (1H, dd, *J*_3,4_ 10.3, *J*_2,3_ 2.8 Hz, H-3), 3.81 (1H, dd, *J*_6a,6b_ 11.7, *J*_6a,5_ 2.6 Hz, H-6a), 3.77 (3H, s, O*Me*), 3.78–3.75 (1H, m, H-6b), 3.72–3.68 (1H, m, H-5), 3.68 (1H, dd, *J*_2,3_ 2.8, *J*_1,2_ 1.3 Hz, H-2), 3.28 (6H, s, 2×O*Me*), 3.27 (3H, s, O*Me*), 1.34 (3H, s, *Me*), 1.29 (3H, s, *Me*).

*δ*_C_ (126 MHz, CDCl_3_) 159.1, 130.7, 129.7, 129.1, 128.2, 113.62, 113.6, 113.5, 100.6 (C-1), 99.8, 99.5, 75.2 (C-2), 72.9, 71.1 (C-5), 69 (C-3), 63.6 (C-4), 61.4 (C-6), 55.2, 54.6, 47.9, 47.8, 17.8, 17.7. *ν*_max_ (KBr) 3060, 3030, 2981, 2950, 1487, 1382, 1216, 1134, 892 cm^−1^. [α]_D_ +132.3 (*c* 1.0, CHCl_3_). *R_f_*=0.28; Tol/EA 30%. HRMS-(TOF): MH^+^, found 429.2128. C_21_H_33_O_9_ requires 429.2125.

#### 4.1.3 Compound **14**

*δ*_H_ (500 MHz, CDCl_3_) 7.41–7.35 (2H, m, CH_2_*Ph*OMe), 7.28–7.23 (2H, m, CH_2_*Ph*OMe), 6.90–6.81 (4H, m, 2×CH_2_*Ph*OMe), 4.88 and 4.63 (2H, AB spectrum, *J* 11.7 Hz, C*H*_2_PhOMe), 4.70 (1H, d, *J*_1,2_ 1.4 Hz, H-1), 4.59 and 4.53 (2H, AB spectrum, *J* 11.6 Hz, C*H*_2_PhOMe), 4.17 (1H, t, *J*_3,4_=*J*_4,5_=10.2 Hz, H-4), 4.07 (1H, dd, *J*_3,4_ 10.2, *J*_3,2_ 2.9 Hz, H-3), 3.90–3.85 (1H, m, H-5), 3.83 (s, 3H, O*Me*), 3.82 (s, 3H, O*Me*), 3.76 (1H, dd, *J*_6a,6b_ 11.1, *J*_6a,5_ 2.2 Hz, H-6a), 3.72 (1H, dd, *J*_6a,6b_ 11.1, *J*_6b,5_ 5.5 Hz, H-6b), 3.68 (1H, dd, *J*_3,2_ 2.9, *J*_1,2_ 1.6 Hz, H-2), 3.35 (3H, s, O*Me*), 3.29 (3H, s, O*Me*), 3.21 (3H, s, O*Me*), 1.35 (3H, s, *Me*), 1.29 (3H, s, *Me*).

*δ*_C_ (126 MHz, CDCl_3_) 159.1, 159, 130.9, 130.7, 129.6, 129.1, 113.6, 100.4 (C-1), 99.8, 99.5, 75.4 (C-2), 73.1, 72.7, 71 (C-5), 69.2 (C-3), 68.6 (C-6), 63.8 (C-4), 55.1, 54.5, 47.83, 47.8, 17.85, 17.8. [α]_D_ +140.6 (*c* 1.0, CHCl_3_). *R_f_*=0.6; Tol/EA 30%. HRMS-(TOF): MH^+^, found 549.2691. C_29_H_41_O_10_ requires 549.2700.

#### 4.1.4 Compound **13** from **14**

A solution of **13** (6.5 g, 11.84 mmol) in MeOH/ethyl acetate 1:1 (120 mL) was hydrogenated under slight positive pressure of H_2_ (balloon) in the presence of Pd/C 20% catalyst (0.6 g) for 3 h at rt. The reaction mixture was filtered through a pad of Celite. The filter cake was washed additionally with MeOH; the combined filtrate was concentrated. The residue was purified by flash chromatography in Tol/EA 5→50% to give 2.56 g (5.98 mmol, 50%) of the title compound **13** and 2.72 g (4.95 mmol, 41%) of the starting material **14**.

#### 4.1.5 (2′*S*,3′*S*)-Methyl 6-deoxy-2-*O*-(4-methoxybenzyl)-3,4-*O*-(2′,3′-dimethoxybutane-2′,3′-diyl)-6-iodo-α-d-mannopyranoside (**15**)

To a solution of **13** (11.37 g, 26.53 mmol) and PPh_3_ (10.23 g, 39 mmol) in toluene (270 mL) imidazole (5.41 g, 79.5 mmol) and iodine (9.13 g, 36 mmol) were sequentially added to form a biphasic mixture with brown coloured lower layer. The reaction was placed into preheated oil bath (70 °C) and stirred for 4 h while the colour gradually faded. The reaction was cooled and concentrated. The residue was dissolved in CHCl_3_, absorbed on silica (80 g) and purified by flash chromatography in PE/EA 0→20% to give 11.13 g (20.67 mmol, 78%) of the title compound **15** as white solid.

*δ*_H_ (500 MHz, CDCl_3_) 7.39–7.35 (2H, m, CH_2_*Ph*OMe), 6.90–6.86 (2H, m, CH_2_*Ph*OMe), 4.87 and 4.62 (2H, AB spectrum, *J* 11.7 Hz, C*H*_2_PhOMe), 4.68 (1H, d, *J*_1,2_ 1.3 Hz, H-1), 4.04 (1H, dd, *J*_3,4_ 10.1, *J*_3,2_ 2.8 Hz, H-3), 3.95 (1H, dd, *J*_3,4_ 10.1, *J*_4,5_ 9.1 Hz, H-4), 3.83 (3H, s, O*Me*), 3.69 (1H, m, H-5), 3.68 (1H, dd, *J*_2,3_ 2.8, *J*_1,2_ 1.3 Hz, H-2), 3.59 (1H, dd, *J*_6a,6b_ 10.5, *J*_6a,5_ 2.2 Hz, H-6a), 3.39 (3H, s, O*Me*), 3.31 (3H, s, O*Me*), 3.29 (3H, s, O*Me*), 3.26 (1H, dd, *J*_6a,6b_ 10.5, *J*_6b,5_ 8.8 Hz, H-6b), 1.36 (3H, s, *Me*), 1.32 (3H, s, *Me*).

*δ*_C_ (126 MHz, CDCl_3_) 159.1, 130.7, 129.6, 113.6, 100.5 (C-1), 99.9, 99.7, 75.2 (C-2), 72.8, 70.7 (C-5), 68.7 (C-3), 67.7 (C-4), 55.3, 54.9, 48.2, 48.1, 17.8, 5.2 (C-6). *ν*_max_ (KBr) 3084, 3052, 2968, 2934, 2863, 1591, 1472, 1430, 988, 763 cm^−1^. *R_f_*=0.35; PE/EA 10%, [α]_D_ 151.5 (*c* 1.00, CHCl_3_). Mp 82.5 °C petroleum ether/chloroform. HRMS-(TOF): MNa^+^, found 561.0977. C_21_H_31_INaO_8_ requires 561.0961.

#### 4.1.6 2-[(*R*)-[(2*S*,3*R*,5*R*,6*R*)-5,6-Dimethoxy-5,6-dimethyl-3-vinyl-1,4-dioxan-2-yl]-[(4-methoxyphenyl)methoxy]methyl]-1*H*-imidazole (**16**)^40^

Zinc dust (13.7 g, 210 mmol) was activated by swirling with 3 M HCl (50 ml) for 3 min. The acid was decanted and the sediment was washed (3×50 ml) with water, then EtOH (50 ml) and finally with ether (50 ml). The light grey powder was dried in vacuum and added to a solution of **15** (11.13 g, 20.67 mmol) in THF/water (10:1) (100 mL). The reaction was brought to reflux (85 °C oil bath) and stirred for 40 min. The reaction was cooled to rt and filtered through a pad of Celite. The filter cake was washed with ether. The combined filtrate was concentrated, diluted with CHCl_3_ and washed with brine. The aqueous layer was extracted additionally with CHCl_3_ twice. The combined organic layer was dried, concentrated and dried briefly in vacuum to give clear oily residue (about 10 g). The residue was dissolved in 7 M methanolic ammonia (100 mL) at 4 °C (ice-bath) and mixed with 40% aqueous glyoxal solution (10 mL). The reaction was removed from the cooling bath and further stirred at rt for 1.5 h while the initially formed thick white precipitate dissolved and solution progressively turned yellow. The reaction was then heated up to 70 °C and kept at this temperature for 2 h; the solution turned brown with brown sediment. The reaction was cooled and volatiles were removed in vacuum. The residue was dissolved in CHCl_3_/MeOH, absorbed on silica (50 g) and purified by flash chromatography in Tol/Me_2_CO 5→30% to give 6.1 g (14.58 mmol, 70%) of the title compound **16** as clear syrup.

*δ*_H_ (500 MHz, CDCl_3_) 7.27–7.21 (2H, m, CH_2_*Ph*OMe), 7.07 (2H, s, H-4′, H-5′), 6.87–6.82 (2H, m, CH_2_*Ph*OMe), 5.69 (1H, ddd, *J*_4,5 trans_ 17.3, *J*_4,5 cis_ 10.3, *J*_3,4_ 7.7 Hz, H-4), 5.24 (2H, m, H-5a, H-5b), 4.60 (1H, d, *J*_1,2_ 2.4, H-1), 4.52–4.45 (2H, m, C*H*_2_PhOMe), 4.12 (1H, dd, *J*_2,3_ 10.4, *J*_1,2_ 2.4 Hz, H-2), 3.79 (3H, s, O*Me*), 3.66 (1H, ddt, *J*_2,3_ 10.4, *J*_3,4_ 7.8, *J*_3,5_ 0.8 Hz, H-3), 3.38 (s, 3H, O*Me*), 3.12 (3H, s, O*Me*), 1.41 (3H, s, *Me*), 1.30 (3H, s, *Me*).

*δ*_C_ (126 MHz, CDCl_3_) 159.1, 144.7 (C-2′), 133.0 (C-4), 130.1, 129.2 (br s, C-4′/5′), 120.9 (C-5), 116.4 (br s, C-4′/5′), 113.7, 98.7, 98.5, 73 (C-2), 72.4 (C-1), 70.7, 70.3 (C-3), 55.2, 48.3, 48, 17.8, 17.5. *ν*_max_ (KBr) 3067, 3033, 2869, 1877, 1812, 1599, 1535, 1497, 1455, 1364, 1343, 1113, 1028, 963 cm^−1^. *R_f_*=0.25; Tol/Me_2_CO 30%, [α]_D_ +41.6 (*c* 0.62, CHCl_3_). HRMS-(TOF): MH^+^, found 419.2185. C_22_H_31_N_2_O_6_ requires 419.2182.

#### 4.1.7 (1*R*)-1-[(2*R*,3*R*,5*R*,6*R*)-3-[(*R*)-1*H*-Imidazol-2-yl-[(4-methoxyphenyl)methoxy]methyl]-5,6-dimethoxy-5,6-dimethyl-1,4-dioxan-2-yl]-2-triisopropylsilyloxy-ethanol (**17**)^40^

Freshly prepared solution of K_3_Fe(CN)_6_ (0.888 g, 2.7 mmol), K_2_CO_3_ (0.373 g, 2.7 mmol) and K_2_OsO_4_·2H_2_O (0.008 g, 0.022 mmol) in *t*-BuOH/water 1:1 (16 mL) was added to a solution of **16** (0.375 g, 0.9 mmol) and methanesulfonamide (0.095 g, 1 mmol) in *t*-BuOH/THF 1:1 (2.7 mL). The resulting slurry was vigorously stirred for 16 h. The reaction was quenched by addition of an excess of Na_2_S_2_O_5_ (1 g), stirred for 0.5 h rt, diluted with water and extracted with ethyl acetate three times. The combined organic layer was dried and concentrated to give 0.41 g (0.9 mmol) of the crude product; the TLC revealed complete consumption of the starting material and formation of a more polar single product (Tol/Me_2_CO 40%; *R_f_*=0.15), (DCM/MeOH 5%; *R_f_*=0.3).

The residue was dissolved in pyridine (5 mL) and treated with triisopropyl chlorosilane (0.213 mL, 1 mmol). The reaction was kept at 50 °C for 16 h. The reaction was cooled to rt, quenched by addition of MeOH (0.5 mL), kept for 20 min at rt and concentrated. The residue was dissolved in DCM and washed with water. The layers were separated; the aqueous layer was extracted with DCM once more. The combined organic layer was dried and concentrated. The residue was purified by flash chromatography in PE/EA 5→50% to give 0.422 g (0.693 mmol, 77%) of the title compound **17** as viscous syrup.

*δ*_H_ (500 MHz, CDCl_3_) 7.28–7.24 (2H, m, CH_2_*Ph*OMe), 7.04 (2H, s, H-4′, H-5′), 6.88–6.83 (2H, m, CH_2_*Ph*OMe), 5.35 (1H, d, *J*_1,2_ 2.9 Hz, H-1), 4.55 (2H, s, C*H*_2_PhOMe), 4.26 (1H, dd, *J*_2,3_ 10.1, *J*_1,2_ 2.9 Hz, H-2), 3.89 (1H, dd, *J*_5a,5b_ 9.9, *J*_5a,4_ 3.6 Hz, H-5a), 3.80 (3H, s, O*Me*), 3.77 (1H, dd, *J*_5a,5b_ 9.9, *J*_5b,4_ 3.4 Hz, H-5b), 3.61 (1H, dt, *J*_4,5_ 8.5, *J*_4,5b_=*J*_4,5b_ 3.5 Hz, H-4), 3.38 (3H, s, O*Me*), 3.34 (1H, dd, *J*_2,3_ 10.1, *J*_3,4_ 8.6, H-3), 3.11 (3H, s, O*Me*), 1.38 (3H, s, *Me*), 1.26 (3H, s, *Me*), 1.11–0.97 (21H, m, Si(C*H*(*Me*)_2_)_3_).

*δ*_C_ (126 MHz, CDCl_3_) 159, 145.8 (C-2′), 130.5, 129.1, 113.7, 98.4, 98.3, 73.3 (C-1), 73.0 (C-2), 71.7 (C-4), 71.1, 67.2 (C-3), 63.1 (C-5), 55.2, 48.3, 48, 17.9, 17.7, 17.4, 11.9. *ν*_max_ (KBr) 3028, 3012, 2989, 2899, 1732, 1455, 1383, 1229, 1159, 1072, 857 cm^−1^. *R_f_*=0.33; PE/EA 40%, [α]_D_ +54.2 (*c* 1.0, CHCl_3_). HRMS-(TOF): MH^+^, found 609.3565. C_31_H_53_N_2_O_8_Si requires 609.3571.

#### 4.1.8 (2*S*,3*S*,4a*S*,5*R*,10*S*,10a*R*)-2,3-Dimethoxy-5-((4-methoxybenzyl)oxy)-2,3-dimethyl-10-(((triisopropylsilyl)oxy)methyl)-2,3,4a,5,10,10a-hexahydro-[1,4]dioxino[2,3-*d*]imidazo[1,2-*a*]pyridine (**18**)^41^

To a solution of **17** (0.057 g, 0.095 mmol) and Py (0.032 mL, 0.38 mmol) in DCM (1.5 mL) cooled to −15 °C trifluoromethanesulfonic anhydride (0.047 mL, 0.28 mmol) was added dropwise. The reaction was allowed to warm-up to the rt and stirred for 1 h at rt. The reaction was diluted with DCM and washed with a mixture of NaHCO_3_ solution and brine. The aqueous layer was extracted with DCM once more. The combined organic layer was dried and concentrated. The residue was purified by flash chromatography Tol/EA 10→40% to give 0.055 g (0.094 mmol, 100%) of the title compound **18** as glassy solid.

*δ*_H_ (500 MHz, CDCl_3_) 7.47–7.41 (2H, m, CH_2_*Ph*OMe), 7.07 (1H, d, *J*_2,3_ 1.3 Hz, H-2), 7.01 (1H, d, *J*_2,3_ 1.3 Hz, H-3), 6.90–6.83 (2H, m, CH_2_*Ph*OMe), 4.95 and 4.78 (2H, AB spectrum, *J* 11.1 Hz, C*H*_2_PhOMe), 4.83 (1H, dd, *J*_6,7_ 11.1, *J*_6,5_ 7.0 Hz, H-6), 4.78 (1H, d, *J*_8,7_ 3.8 Hz, H-8), 4.39 (1H, dd, *J*_7,6_ 11.1, *J*_7,8_ 3.8 Hz, H-7), 4.34–4.29 (1H, m, H-5), 4.12 (1H, dd, *J*_5*a,5*b_ 10.5, *J*_5*a,5_ 3.0 Hz, H-5^∗^a), 3.98 (1H, dd, *J*_5*a,5*b_ 10.6, *J*_5*b,5_ 5.1 Hz, H-5^∗^b), 3.80 (3H, s, O*Me*), 3.32 (3H, s, O*Me*), 3.25 (3H, s, O*Me*), 1.41 (3H, s, *Me*), 1.34 (3H, s, *Me*), 1.10–0.97 (21H, m, Si(C*H*(*Me*)_2_)_3_).

*δ*_C_ (126 MHz, CDCl_3_) 158.9, 143.5 (C-8a), 131, 129.5, 129.2 (C-2), 119.1 (C-3), 113.54, 113.5, 113.4, 99.4, 99.3, 72.0, 70.1 (C-8), 67.2 (C-7), 62.8 (C-6), 62.5 (C-5^∗^), 58.2 (C-5), 55.2, 48.1, 48.03, 18.01, 17.9, 17.6, 11.8. *ν*_max_ (KBr) 3090, 3067, 1952, 1879, 1810, 1751, 1603, 1525, 1496, 1310, 1085, 1028, 980 cm^−1^. *R_f_*=0.3; Tol/EA 30%, [α]_D_ −27.6 (*c* 1.0, CHCl_3_). HRMS-(TOF): MH+, found 591.3460. C_31_H_51_N_2_O_7_Si requires 591.3466.

#### 4.1.9 (1*S*)-1-[(2*R*,3*R*,5*R*,6*R*)-3-[(*R*)-1*H*-Imidazol-2-yl-[(4-methoxyphenyl)methoxy]methyl]-5,6-dimethoxy-5,6-dimethyl-1,4-dioxan-2-yl]-2-triisopropylsilyloxy-ethanol (**19**)^40^

To a solution of DMSO (0.97 mL, 13.7 mmol) in DCM (65 mL) trifluoroacetic anhydride (1.43 mL, 10.25 mmol) was added dropwise at −60 °C. The reaction was stirred for 20 min then a solution of **17** (5.2 g, 8.54 mmol) in DCM (20 mL) was added via capillary. The reaction was further stirred for 45 min before Et_3_N (4.76 mL, 34.16 mmol) was added dropwise. The reaction was stirred for 5 min at −60 °C and then was warmed to rt. The reaction was quenched by addition of 10% citric acid solution and diluted with DCM. The layers were separated; the organic layer was successively washed with water and a mixture of saturated NaHCO_3_ solution and brine. The aqueous layers were additionally extracted with DCM; the combined organic layer was dried and concentrated to yield a semi-solid residue (5.25 g); TLC PE/EA 50% showed disappearance of the starting material (*R_f_*=0.45) and formation of more polar product (*R_f_*=0.35).

The residue was dissolved in EtOH (60 mL) and treated with NaBH_4_ (0.37 g, 10 mmol) at rt for 16 h. The reaction was quenched by addition of acetic acid and concentrated. The residue was partitioned between 1 M HCl and DCM and the layers were separated. The organic layer was washed successively with water and a mixture of saturated NaHCO_3_ solution and brine. The aqueous layers were additionally extracted with DCM. The combined organic layer was dried and concentrated. The residue was purified by flash chromatography in Tol/EA 20→50% to give 0.53 g (0.88 mmol, 9%) of the starting material **17** and 4.8 g (7.9 mmol, 81%) of the title compound **19** as viscous syrup.

*δ*_H_ (500 MHz, CDCl_3_) 7.29–7.24 (2H, m, CH_2_*Ph*OMe), 7.03 (2H, s, H-4′, H-5′), 6.88–6.81 (2H, m, CH_2_*Ph*OMe), 4.77 (1H, d, *J*_1,2_ 2.6 Hz, H-1), 4.66 and 4.58 (2H, AB spectrum, *J* 11.5 Hz, C*H*_2_PhOMe), 4.66 (1H, dd, *J*_2,3_ 10.4, *J*_2,1_ 2.6 Hz, H-2), 3.80 (3H, s, O*Me*), 3.74–3.64 (3H, m, H-3, H-5a, H-5b), 3.53 (1H, ddd, *J* 7.7, 6.3, 1.4 Hz, H-4), 3.35 (3H, s, O*Me*), 3.16 (3H, s, O*Me*), 1.38 (3H, s, *Me*), 1.31 (3H, s, *Me*), 1.07–0.94 (21H, m, Si(C*H*(*Me*)_2_)_3_).

*δ*_C_ (126 MHz, CDCl_3_) 159.1, 145.1 (C-2′/C3′), 130.3, 129.2, 113.7, 98.7, 73.2 (C-1), 71.3, 69.94 (C-4/C-2), 69.9 (C-4/C-2), 66.6 (C-3), 62.7 (C-5), 55.2, 48.2, 47.95, 17.95, 17.8, 17.4, 11.8. *ν*_max_ (KBr) 3030, 3015, 2993, 2899, 1735, 1455, 1383, 1230, 1161, 1072, 855 cm^−1^. *R_f_*=0.35; Tol/EA 40%, [α]_D_ +68.6 (*c* 1.0, CHCl_3_). HRMS-(TOF): MH^+^, found 609.3562. C_31_H_53_N_2_O_8_Si requires 609.3571.

#### 4.1.10 (2*S*,3*S*,4a*S*,5*R*,10*R*,10a*R*)-2,3-Dimethoxy-5-((4-methoxybenzyl)oxy)-2,3-dimethyl-10-(((triisopropylsilyl)oxy)methyl)-2,3,4a,5,10,10a-hexahydro-[1,4]dioxino[2,3-*d*]imidazo[1,2-*a*]pyridine (**20**)^41^

To a solution of **19** (4.57 g, 7.5 mmol) and pyridine (2.42 mL, 30 mmol) in C_2_H_4_Cl_2_ (75 mL) cooled to −15 °C trifluoromethanesulfonic anhydride (3.8 mL, 22.58 mmol) was added dropwise. The reaction was warmed to rt. After 10 min the reaction was placed into preheated oil bath (50 °C) and stirred for 3 h. The reaction was cooled down, diluted with DCM and washed with a mixture of NaHCO_3_ solution and brine. The aqueous layer was extracted with DCM once more. The combined organic layer was dried and concentrated. The residue was purified by flash chromatography Tol/EA 10→40% to give 4.29 g (7.26 mmol, 97%) of the title compound **20** as clear syrup.

*δ*_H_ (500 MHz, CDCl_3_) 7.31–7.26 (2H, m, CH_2_*Ph*OMe), 7.24 (1H, d, *J*_2,3_ 1.2 Hz, H-2), 6.99 (1H, d, *J*_2,3_ 1.2 Hz, H-3), 6.79–6.73 (2H, m, CH_2_*Ph*OMe), 4.77 and 4.65 (2H, AB spectrum, *J* 11.4 Hz, C*H*_2_PhOMe), 4.69 (1H, d, *J*_8,7_ 3.2 Hz, H-8), 4.35 (1H, dd, *J*_6,7_ 10.5, *J*_6,5_ 9.2 Hz, H-6), 4.13 (1H, dd, *J*_5*a,5*b_ 10.7, *J*_5*a,5_ 1.9 Hz, H-5^∗^a), 3.96 (1H, ddd, *J*_5,6_ 9.1, *J*_5,5*b_ 7.2, *J*_5,5*a_ 1.9 Hz, H-5), 3.92 (1H, dd, *J*_7,6_ 10.6, *J*_7,8_ 3.3 Hz, H-7), 3.84 (1H, dd, *J*_5*b,5*a_ 10.7, *J*_5*b,5_ 7.2 Hz, H-5^∗^b), 3.71 (3H, s, O*Me*), 3.21 (3H, s, O*Me*), 3.19 (3H, s, O*Me*), 1.32 (3H, s, *Me*), 1.27 (3H, s, *Me*), 1.07–0.87 (21H, m, Si(C*H*(*Me*)_2_)_3_).

*δ*_C_ (126 MHz, CDCl_3_) 158.9, 143.2 (C-8a), 130.8, 129.5 (C-3), 129.2, 118.8 (C-2), 113.4, 99.5, 99.3, 71.3, 69.7 (C-8), 69.4 (C-7), 64.6 (C-5^∗^), 62.5 (C-6), 60.6 (C-5), 55.2, 48.2, 48.1, 17.9, 17.6, 17.7, 11.8. *ν*_max_ (KBr) 3088, 3060, 1946, 1880, 1801, 1751, 1603, 1525, 1499, 1310, 1083, 1034, 982 cm^−1^. *R_f_*=0.38; Tol/EA 40%, [α]_D_ +40.4 (*c* 1.0, CHCl_3_). HRMS-(TOF): MH^+^, found 591.3465. C_31_H_51_N_2_O_7_Si requires 591.3466.

#### 4.1.11 (*S*)-1-((2*R*,3*R*,5*S*,6*S*)-3-((*R*)-(4,5-Diiodo-1*H*-imidazol-2-yl)((4-methoxybenzyl)oxy)methyl)-5,6-dimethoxy-5,6-dimethyl-1,4-dioxan-2-yl)-2-((triisopropylsilyl)oxy)ethanol (**21**)^40^

To a solution of **19** (0.121 g, 0.2 mmol) in MeCN (2 mL) NIS (0.099 g, 0.44 mmol) was added at rt. The reaction was further stirred for 40 min in the dark. The reaction was diluted with DCM and washed with 0.5 M aqueous sodium thiosulfate (Na_2_S_2_O_3_) solution. The aqueous layer was extracted with DCM once more. The combined organic layer was dried and concentrated. The residue was absorbed on silica (2 g) and purified by flash column chromatography in Tol/EA gradient 5→20% to give 0.133 g (0.15 mmol, 77%) of the title compound **21** as foam.

*δ*_H_ (500 MHz, CDCl_3_) 7.28–7.23 (2H, m, CH_2_*Ph*OMe), 6.88–6.83 (2H, m, CH_2_*Ph*OMe), 4.64 and 4.6 (2H, AB spectrum, *J* 11.4 Hz, C*H*_2_PhOMe), 4.62 (1H, d, *J*_1,2_ 2.6 Hz, H-1), 4.58 (1H, dd, *J*_2,3_ 10.8, *J*_2,1_2.5 Hz, H-2), 3.80 (3H, s, O*Me*), 3.70 (1H, dd, *J*_5a,5b_ 9.2, *J*_5a,4_ 6.2 Hz, H-5a), 3.65 (1H, t, *J*_5a,5b_=*J*_5b,5_=9.3 Hz, H-5b), 3.60 (1H, dd, *J*_3,2_ 10.8, *J*_3,4_1.2 Hz, H-3), 3.56–3.47 (1H, m, H-4), 3.36 (3H, s, O*Me*), 3.23 (3H, s, O*Me*), 2.46 (1H, br d, O*H*-4), 1.41 (3H, s, *Me*), 1.33 (3H, s, *Me*), 1.09–0.86 (21H, m, Si(C*H*(*Me*)_2_)_3_).

*δ*_C_ (126 MHz, CDCl_3_) 159.1, 150.7 (C-2′), 129.9, 129.2, 113.7, 98.7, 98.5, 94.6 (br s, C-4′/5′), 75.9 (br s, C-4′/5′), 72.2 (C-1), 71.3, 70.1 (C-4), 69.4 (C-2), 65.7 (C-3), 62.4 (C-5), 55.3, 48.5, 48.3, 18.0, 17.9, 17.8, 17.2, 11.8. *R_f_*=0.18; Tol/EA 10%. [α]_D_ −13.3 (*c* 1.00, CHCl_3_). HRMS-(TOF): MH^+^, found 861.1510. C_31_H_51_I_2_N_2_O_8_Si requires 861.1504.

#### 4.1.12 (2*S*,3*S*,4a*S*,5*R*,10*R*,10a*R*)-7,8-Diiodo-2,3-dimethoxy-5-((4-methoxybenzyl)oxy)-2,3-dimethyl-10-(((triisopropylsilyl)oxy)methyl)-2,3,4a,5,10,10a-hexahydro-[1,4]dioxino[2,3-*d*]imidazo[1,2-*a*]pyridine (**22**)^41^

A solution of **20** (3.2 g, 5.4 mmol) in DMF (60 mL) was treated with NIS (12.1 g, 53.78 mmol) at 80 °C for 36 h. The reaction was cooled, diluted with ethyl acetate and washed with 0.5 M sodium thiosulfate solution. The layers were separated. The aqueous layer was extracted with ethyl acetate once more. The combined organic layer was dried and concentrated. The residue was absorbed on silica (60 g) and purified by flash chromatography PE/EE 5→30% to give 3.3 g (3.9 mmol, 73%) of the title compound **22** as yellowish foam.

#### 4.1.13 With NIS/PPTS

A solution of **20** (0.296 g, 0.5 mmol), NIS (0.338 g, 0.35 mmol) and pyridinium *p*-toluenesulfonate (0.125 g, 0.11 mmol) in MeCN (5 mL) was stirred at 80 °C for 1 h. The reaction was cooled, diluted with ethyl acetate and washed with 0.5 M sodium thiosulfate solution. The aqueous layer was extracted with ethyl acetate once more. The combined organic layer was dried and concentrated. The residue was purified by flash chromatography PE/EE 5→30% to give 0.259 g (0.31 mmol, 62%) of the title compound **22**.

*δ*_H_ (500 MHz, CDCl_3_) 7.28–7.23 (2H, m, CH_2_*Ph*OMe), 6.78–6.74 (2H, m, CH_2_*Ph*OMe), 4.94 (1H, dd, *J*_6,7_ 10.7, *J*_6,5_ 6.5 Hz, H-6), 4.69 and 4.59 (2H, AB spectrum, *J* 11.4 Hz, C*H*_2_PhOMe), 4.61 (1H, d, *J*_8,7_ 2.8 Hz, H-8), 4.26 (1H, dd, *J*_5*a,5*b_ 10.7, *J*_5*a,5_ 4.8 Hz, H-5^∗^a), 4.03 (1H, ddd, *J*_5,6_ 6.7, *J*_5,5*a_ 4.8, *J*_5,5*b_ 2.5 Hz, H-5), 3.87 (1H, dd, *J*_5*a,5*b_ 10.7, *J*_5*b,5_ 2.5 Hz, H-5^∗^b), 3.80 (1H, dd, *J*_7,6_ 10.6, *J*_7,8_ 2.8 Hz, H-7), 3.73 (3H, s, O*Me*), 3.26 (3H, s, O*Me*), 3.15 (3H, s, O*Me*), 1.31 (3H, s, *Me*), 1.26 (3H, s, *Me*), 0.88–0.77 (21H, m, Si(C*H*(*Me*)_2_)_3_).

*δ*_C_ (126 MHz, CDCl_3_) 158.9, 150 (C-8a), 130.3, 129.6, 113.4, 99.8, 99.6, 97.4 (C-3), 80.1 (C-2), 71.4, 70.1 (C-8), 68.9 (C-7), 63.6 (C-6), 62.9 (C-5^∗^), 62.1 (C-5), 55.3, 48.2, 48.1, 17.8, 17.7, 17.6, 11.8. *ν*_max_ (KBr) 3063, 2865, 1880, 1812, 1613, 1501, 1454, 1352, 1173, 1100, 1031, 911 cm^−1^. *R_f_*=0.3; PE/EE 30%, [α]_D_ −28.5 (*c* 1.0, CHCl_3_). HRMS-(TOF): MH^+^, found 843.1421. C_31_H_49_I_2_N_2_O_7_Si requires 843.1399.

#### 4.1.14 (2*S*,3*S*,4a*S*,5*R*,10*R*,10a*R*)-7-Iodo-2,3-dimethoxy-5-((4-methoxybenzyl)oxy)-2,3-dimethyl-10-(((triisopropylsilyl)oxy)methyl)-2,3,4a,5,10,10a-hexahydro-[1,4]dioxino[2,3-*d*]imidazo[1,2-*a*]pyridine (**23**)^41^

To a solution of **22** (2.02 g, 2.39 mmol) in THF (25 mL) a stock 1 M solution of EtMgBr in THF (3 mL, 3 mmol) was added at 4 °C (ice-bath). After 10 min the reaction was quenched with saturated NH_4_Cl solution. The reaction mixture was diluted with ethyl acetate and brine and the layers were separated. The aqueous layer was extracted with ethyl acetate once more. The combined organic layer was dried and concentrated. The residue was absorbed on silica (15 g) and purified by flash chromatography PE/EA 5→15% to give 1.438 g (2.06 mmol, 84%) of the title compound **23** as foam.

*δ*_H_ (500 MHz, CDCl_3_) 7.39 (1H, s, H-3), 7.37–7.32 (2H, m, CH_2_*Ph*OMe), 6.87–6.81 (2H, m, CH_2_*Ph*OMe), 4.86 and 4.71 (2H, AB spectrum, *J* 11.2 Hz, C*H*_2_PhOMe), 4.75 (1H, d, *J*_8,7_ 3.2 Hz, H-8), 4.42 (1H, dd, *J*_6,7_ 10.6, *J*_6,5_ 9.2 Hz, H-6), 4.16 (1H, dd, *J*_5*a,5*b_ 10.8, *J*_5*a,5_ 2.0 Hz, H-5^∗^a), 4.04 (1H, ddd, *J*_5,6_ 9.1, *J*_5,5*b_ 7.2, *J*_5,5*a_ 2.0 Hz, H-5), 3.98 (1H, dd, *J*_7,6_ 10.6, *J*_7,8_ 3.2 Hz, H-7), 3.87 (1H, dd, *J*_5*a,5*b_ 10.7, *J*_5*b,5_ 7.1 Hz, H-5^∗^b), 3.79 (3H, s, O*Me*), 3.29 (3H, s, O*Me*), 3.27 (3H, s, O*Me*), 1.40 (3H, s, *Me*), 1.34 (3H, s, *Me*), 1.13–1.01 (21H, m, Si(C*H*(*Me*)_2_)_3_).

*δ*_C_ (126 MHz, CDCl_3_) 158.9, 145.4 (C-8a), 130.6, 129.5, 124.6 (C-3), 113.4, 99.6, 99.3, 82.2 (C-2), 71.8, 69.6 (C-8), 69.2 (C-7), 64.5 (C-5^∗^), 62.2 (C-6), 60.8 (C-5), 55.2, 48.2, 48.1, 17.9, 17.8, 11.8. *ν*_max_ (KBr) 3063, 2874, 1875, 1734, 1495, 1453, 1368, 1257, 1112, 1023, 943 cm^−1^. *R_f_*=0.38; PE/EA 15%, [α]_D_ +53.2 (*c* 1.0, CHCl_3_). HRMS-(TOF): MH^+^, found 717.2435. C_31_H_50_IN_2_O_7_Si requires 717.2432.

#### 4.1.15 (2*S*,3*S*,4a*R*,5*R*,11*S*,11a*S*)-7,8,11-Triiodo-2,3-dimethoxy-5-((4-methoxybenzyl)oxy)-2,3-dimethyl-3,4a,5,10,11,11a-hexahydro-2*H*-[1,4]dioxino[2,3-*d*]imidazo[1,2-*a*]azepine (**25**)^42^

To a stirred solution of **16** (0.063 g, 0.15 mmol) in MeCN (3 mL) NIS (0.111 g, 0.5 mmol) was added at rt. The reaction was further stirred for 24 h in the dark. The reaction was diluted with DCM and washed with 0.5 M sodium thiosulfate solution. The aqueous layer was extracted with DCM once more. The combined organic layer was dried and concentrated. The residue was purified by flash column chromatography in PE/EA 5→15% to give 0.093 g (0.12 mmol, 78%) of the title compound **25** as viscous syrup.

*δ*_H_ (500 MHz, CDCl_3_) 7.15–7.10 (2H, m, CH_2_*Ph*OMe), 6.83–6.78 (2H, m, CH_2_*Ph*OMe), 4.80 (1H, d, *J*_9,8_ 1.4 Hz, H-9), 4.77 and 4.43 (2H, AB spectrum, *J* 11.9 Hz, C*H*_2_PhOMe), 4.50 (1H, dd, *J*_5a,5b_ 14.6, *J*_5a,6_ 2.8 Hz, H-5a), 4.43 (1H, dd, *J*_5a,5b_ 14.6, *J*_5b,6_ 11.4 Hz, H-5b), 4.31 (1H, dd, *J*_7,6_ 10.8, *J*_7,8_ 9.2 Hz, H-7), 3.86 (1H, br dt, *J*_6,5b_ 11.4, *J*_6,5a_ 2.8 Hz, H-6), 3.81 (3H, s, O*Me*), 3.66 (1H, dd, *J*_8,7_ 9.2, *J*_8,9_ 1.4 Hz, H-8), 3.43 (3H, s, O*Me*), 3.18 (3H, s, O*Me*), 1.36 (3H, s, *Me*), 1.31 (3H, s, *Me*).

*δ*_C_ (126 MHz, CDCl_3_) 159.2, 149.3 (C-9a), 129.7, 129.2, 113.6, 113.5, 100.29, 100.0, 99.9, 94.3 (C-2), 85.5 (C-3), 76.5 (C-9), 72.6, 72.1 (C-7 and C-8), 72.0, 55.3 (C-5), 54.2, 49.1, 48.3, 26.7 (C-6), 17.4, 17.2. *R_f_*=0.32; PE/EA 15%, [α]_D_ +3.4 (*c* 1.0, CHCl_3_). HRMS-(TOF): MH^+^, found 796.9078. C_22_H_28_I_3_N_2_O_6_ requires 796.9082.

#### 4.1.16 (2*S*,3*S*,4a*S*,5*R*,10*R*,10a*R*)-2,3-Dimethoxy-5-((4-methoxybenzyl)oxy)-2,3-dimethyl-7-(phenylethynyl)-10-(((triisopropylsilyl)oxy)methyl)-2,3,4a,5,10,10a-hexahydro-[1,4]dioxino[2,3-*d*]imidazo[1,2-*a*]pyridine (**26**)^41^

A solution of **23** (1.43 g, 2.0 mmol), phenylacetylene (1.1 mL, 10 mmol) and Et_3_N (1.4 mL, 10 mmol) in DMF (20 mL) was degassed by freezing, evacuating and thawing three times. Cuprous iodide (0.038 g, 0.2 mmol) and Pd(PPh_3_)_4_ (0.23 g, 0.2 mmol) were then added to the reaction flask. The reaction was placed in the preheated (80 °C) oil bath stirred for 16 h. The reaction was cooled and concentrated. The residue was dissolved in ethyl acetate and washed with water and brine. The aqueous layer was extracted with ethyl acetate once more. The combined organic layer was dried and concentrated. The brown residue was purified by flash column chromatography in PE/EA 10→20% to give 1.28 g (1.86 mmol, 93%) of the title compound **26** as amber amorphous solid.

*δ*_H_ (500 MHz, CDCl_3_) 7.56 (1H, s, H-3), 7.55–7.52 (2H, m, CH_2_*Ph*OMe), 7.41–7.36 (2H, m, *Ph*), 7.35–7.30 (3H, m, *Ph*), 6.88–6.82 (2H, m, CH_2_*Ph*OMe), 4.90 and 4.80 (2H, AB spectrum, *J* 11.2 Hz, C*H*_2_PhOMe), 4.87–4.83 (1H, br d, H-8), 4.43 (1H, dd, *J*_6,7_ 10.5, *J*_6,5_ 9.2, Hz, H-6), 4.20 (1H, dd, *J*_5*a,5*b_ 10.8, *J*_5*a,5_ 1.9 Hz, H-5^∗^a), 4.06 (1H, ddd, *J*_5,6_ 9.3, *J*_5,5*b_ 7.3, *J*_5,5*a_ 1.9 Hz, H-5), 4.03 (1H, dd, *J*_7,6_ 10.6, *J*_7,8_ 3.2 Hz, H-7), 3.90 (1H, dd, *J*_5*a,5*b_ 10.8, *J*_5*b,5_ 7.4 Hz, H-5^∗^b), 3.81 (3H, s, O*Me*), 3.30 (3H, s, O*Me*), 3.30 (3H, s, O*Me*), 1.42 (3H, s, *Me*), 1.36 (3H, s, O*Me*), 1.17–1.04 (21H, m, Si(C*H*(*Me*)_2_)_3_).

*δ*_C_ (126 MHz, CDCl_3_) 158.9, 143.7 (C-8a), 131.6, 130.7, 129.6, 128.2, 128.0, 124.4 (C-2), 123.3, 122.8 (C-3), 113.4, 99.6, 99.3, 89.3 (C-2″), 82.9 (C-2′), 71.8, 69.9 (C-8), 69.2 (C-7), 64.7 (C-5^∗^), 62.2 (C-6), 61.0 (C-5), 55.2, 48.2, 48.1, 18.0, 17.9, 17.72, 17.7, 11.8. *ν*_max_ (KBr) 3089, 2941, 2100, 1599, 1496, 1454, 1364, 1301, 1174, 1112, 1050, 1028, 913 cm^−1^. *R_f_*=0.25; PE/EA 15%, [α]_D_ +91.4 (*c* 1.0, CHCl_3_). HRMS-(TOF): MH^+^, found 691.3774. C_39_H_55_N_2_O_7_Si requires 691.3779.

#### 4.1.17 (2*S*,3*S*,4a*R*,5*R*,10*R*,10a*R*)-2,3-Dimethoxy-2,3-dimethyl-7-(phenylethynyl)-10-(((triisopropylsilyl)oxy)methyl)-2,3,4a,5,10,10a-hexahydro-[1,4]dioxino[2,3-*d*]imidazo[1,2-*a*]pyridin-5-ol (**27**)^41^

A solution of **26** (0.266 g, 0.38 mmol) in DCM/water (20:1; 5 mL) DDQ (0.15 g, 0.67 mmol) was added in one portion. The reaction was stirred for 4 h at rt. The reaction was quenched by addition of sodium metabisulfite (Na_2_S_2_O_5_) solution, diluted with DCM and washed successively with sodium metabisulfite (Na_2_S_2_O_5_) solution and a mixture of concentrated NaHCO_3_ solution and brine. The aqueous layer was extracted with DCM once more. The combined organic layer was dried and evaporated. The residue was purified by flash column chromatography in Tol/EA 5→50% to give 0.173 g (0.3 mmol, 80%) of the title compound **27**.

*δ*_H_ (500 MHz, CDCl_3_) 7.55 (1H, s, H-3), 7.54–7.50 (2H, m, *Ph*), 7.34–7.27 (3H, m, *Ph*), 5.08 (1H, d, *J*_8,7_ 3.4 Hz, H-8), 4.46 (1H, br s, O*H*-8), 4.38 (1H, dd, *J*_6,7_ 10.5, *J*_6,5_ 9.5 Hz, H-6), 4.24 (1H, dd, *J*_5*a,5*b_ 10.8, *J*_5*a,5_ 1.6 Hz, H-5^∗^a), 4.06 (1H, ddd, *J*_5,6_ 9.2, *J*_5,5*b_ 7.4, *J*_5,5*a_ 1.6 Hz, H-5), 4.01–3.94 (2H, m, H-5^∗^b, H-7), 3.30 (3H, s, O*Me*), 3.27 (3H, s, O*Me*), 1.40 (3H, s, *Me*), 1.34 (3H, s, *Me*), 1.16–1.03 (21H, m, Si(C*H*(*Me*)_2_)_3_).

*δ*_C_ (126 MHz, CDCl_3_) 144.7 (C-8a), 131.5, 128.2, 128, 124.3, 123.4, 122.4 (C-3), 100.0, 99.9, 99.4, 89.2 (C-2″), 83.0 (C-2′), 68.5 (C-7), 64.5 (C-5^∗^), 63.2 (C-8), 61.8 (C-6), 60.8 (C-5), 48.2, 48.1, 17.9, 17.9, 17.7, 17.6, 11.8. *ν*_max_ (KBr) 3089, 2945, 1951, 1731, 1686, 1602, 1534, 1496, 1384, 1263, 1092, 1028, 909 cm^−1^. *R_f_*=0.2; Tol/EA 40%, [α]_D_ +80.3 (*c* 1.0, CHCl_3_). HRMS-(TOF): MH^+^, found 571.3209. C_31_H_47_N_2_O_6_Si requires 571.3203.

#### 4.1.18 (2*S*,3*S*,4a*R*,5*S*,10*R*,10a*R*)-5-Azido-2,3-dimethoxy-2,3-dimethyl-7-(phenylethynyl)-10-(((triisopropylsilyl)oxy)methyl)-2,3,4a,5,10,10a-hexahydro-[1,4]dioxino[2,3-*d*]imidazo[1,2-*a*]pyridine (**28**)^41^

To a stirred solution of **27** (0.648 g, 1.14 mmol) in toluene (20 mL) diphenylphosphoryl azide (1.23 mL, 5.7 mmol) followed by DBU (0.852 mL, 5.7 mmol) was added at rt. The reaction was placed in a preheated oil bath (80 °C) and kept for 2 h. The reaction was cooled and concentrated. The brown residue was absorbed on silica and purified by flash chromatography PE/EE 5→30% to give 0.574 g (0.96 mmol, 85%) of the title compound **28** as foam.

*δ*_H_ (500 MHz, CDCl_3_) 7.55–7.51 (2H, m, *Ph*), 7.47 (1H, s, H-3), 7.37–7.30 (3H, m, *Ph*), 4.73 (1H, d, *J*_8,7_ 9.5 Hz, H-8), 4.29 (1H, d, *J* 9.7 Hz, H-5^∗^a), 4.09–3.93 (4H, m, H-5, H-5^∗^b, H-6, H-7), 3.38 (3H, s, O*Me*), 3.30 (3H, s, O*Me*), 1.42 (3H, s, *Me*), 1.37 (3H, s, *Me*), 1.17–1.04 (21H, m, Si(C*H*(*Me*)_2_)_3_).

*δ*_C_ (126 MHz, CDCl_3_) 141.7 (C-8a), 131.5, 128.2, 128.1, 125.3 (C-2), 123.2, 121.4 (C-3), 99.65, 99.6, 89.4 (C-2″), 82.7 (C-2′), 70.6 (C-7), 64.3, 62.4 (C-5^∗^), 59.7, 57.5 (C-8), 48.4, 48.3, 17.9, 17.8, 17.6, 17.4, 11.9. *ν*_max_ (KBr) 3089, 3032, 2950, 2835, 2109, 1454, 1407, 1362, 1332, 1269, 1145, 1098, 1018, 917 cm^−1^. *R_f_*=0.26; PE/EE 20%, [α]_D_ +98.7 (*c* 0.51, CHCl_3_). HRMS-(TOF): MH^+^, found 596.3275. C_31_H_46_N_5_O_5_Si requires 596.3268.

#### 4.1.19 *N*-((2*S*,3*S*,4a*R*,5*S*,10*R*,10a*R*)-2,3-Dimethoxy-2,3-dimethyl-7-phenethyl-10-(((triisopropylsilyl)oxy)methyl)-2,3,4a,5,10,10a-hexahydro-[1,4]dioxino[2,3-*d*]imidazo[1,2-*a*]pyridin-5-yl)acetamide (**29**)^41^

A solution of **28** (0.045 g, 0.076 mmol) in ethyl acetate (2 mL) was stirred under slight positive pressure of H_2_ (balloon) in the presence of 20% Pd_2_(OH)_2_ on carbon (0.025 g) for 40 min. The reaction was filtered through a pad of Celite and concentrated. The residue was dissolved in DCM (2 mL) and treated with an excess of acetic anhydride (0.05 mL), and Et_3_N (0.2 mL) for 2 h at rt. The reaction was quenched with MeOH (0.1 mL), stirred for 20 min, diluted with DCM and washed successively with 1 M HCl, water and a mixture of saturated NaHCO_3_ solution and brine. The aqueous layers were additionally extracted with DCM. The combined organic layer was dried and concentrated. The residue was purified by flash chromatography Tol/Me_2_CO 5→40% to give 0.047 g (0.071 mmol, 93%) of the title compound **29** as foam.

*δ*_H_ (500 MHz, CDCl_3_) 7.21–7.16 (2H, m, *Ph*), 7.14–7.07 (3H, m, *Ph*), 6.89 (1H, s, H-3), 4.50 (1H, t, *J*_7,6_=*J*_7,8_=10.0 Hz, H-7), 4.43–4.34 (1H, br t, H-8), 4.21 (1H, dd, *J*_5*a,5*b_ 11.0, *J*_5*a,5_ 1.7 Hz, H-5^∗^a), 4.03 (1H, ddd, *J*_5,6_ 9.8, *J*_5*b,5_ 5.8, *J*_5*a,5_ 1.7 Hz, H-5), 3.91 (1H, dd, *J*_5*a,5*b_ 11.0, *J*_5*b,5_ 5.8 Hz, H-5^∗^b), 3.72 (1H, t, *J*_6,7_=*J*_6,5_=10 Hz, H-6), 3.16 (3H, s, O*Me*), 3.07 (3H, s, O*Me*), 2.99–2.91 (1H, m, H-2′a), 2.83–2.70 (3H, m, H-2′b, H-2″a, H-2″b), 1.99 (3H, s, COC*H*_3_), 1.21 (3H, s, *Me*), 1.12 (3H, s, *Me*), 1.08–0.90 (21H, m, Si(C*H*(*Me*)_2_)_3_).

*δ*_C_ (126 MHz, CDCl_3_) 170.4 (*C*OCH_3_), 143.6 (C-8a), 142.2, 141.6, 128.4, 128.3, 125.8, 113.6 (C-3), 99.4, 99.2, 67.9 (C-7), 65.8 (C-6), 62.7 (C-5^∗^), 59.14 (C-5), 49.5 (C-8), 48.1, 47.7, 35.8 (C-2′), 30.6 (C-2″), 23.4 (CO*C*H_3_), 18.0, 17.9, 17.6, 17.5, 12.0. *R_f_*=0.22; Tol/Me_2_CO 30%, [α]_D_ +105.3 (*c* 1.0, CHCl_3_). HRMS-(TOF): MH^+^, found 616.3775. C_33_H_54_N_3_O_6_Si requires 616.3782.

#### 4.1.20 *N*-((2*S*,3*S*,4a*R*,5*S*,10*R*,10a*R*)-2,3-Dimethoxy-2,3-dimethyl-7-phenethyl-10-(((triisopropylsilyl)oxy)methyl)-2,3,4a,5,10,10a-hexahydro-[1,4]dioxino[2,3-*d*]imidazo[1,2-*a*]pyridin-5-yl)propionamide (**30**)^41^

Prepared from **28** in 83% yield as described for preparation of **29** with replacement of acetic anhydride for propionic anhydride.

*δ*_H_ (500 MHz, CDCl_3_) 7.22–7.15 (2H, m, *Ph*), 7.14–7.06 (3H, m, *Ph*), 6.88 (1H, s, H-3), 4.53–4.38 (2H, m, H-7, H-8), 4.21 (1H, d, *J*_5*a,5*b_ 10.9 Hz, H-5^∗^a), 4.04 (1H, dd, *J*_5,6_ 9.6, *J*_5*b,5_ 5.8 Hz, H-5), 3.90 (1H, dd, *J*_5*a,5*b_ 11.0, *J*_5*b,5_ 5.9 Hz, H-5^∗^b), 3.73 (1H, t, *J*_6,5_=*J*_6,7_=9.9 Hz, H-6), 3.16 (3H, s, O*Me*), 3.08 (3H, s, O*Me*), 2.92 (1H, dt, *J*_2′a_, _2′b_ 13.8, *J* 7.1 Hz, H-2′a), 2.84–2.70 (3H, m, H-2′b, H-2″a, H-2″b), 2.23 (2H, m, COC*H*_2_CH_3_), 1.22 (4H, s, *Me*), 1.15 (3H, s, *Me*), 1.06 (4H, t, *J* 7.5 Hz, COCH_2_C*H*_3_), 1.04–0.93 (21H, m, Si(C*H*(*Me*)_2_)_3_).

*δ*_C_ (126 MHz, CDCl_3_) 174.1 (*C*OCH_2_CH_3_), 143.5 (C-8a), 142.1, 141.4, 128.4, 128.3, 125.8, 113.6 (C-3), 99.3, 99.2, 68.0 (C-7), 65.7 (C-6), 62.7 (C-5^∗^), 59.2 (C-5), 49.3 (C-8), 48.1, 47.7, 35.7 (C-2′), 30.4 (C-2″), 29.6 (CO*C*H_2_CH_3_), 18.0, 17.95, 17.6, 17.4, 12.0, 9.8 (COCH_2_*C*H_3_). *R_f_*=0.35; Tol/Me_2_CO 30%, [α]_D_ +103.5 (*c* 1.0, CHCl_3_). HRMS-(TOF): MH^+^, found 630.3931. C_34_H_56_N_3_O_6_Si requires 630.3938.

#### 4.1.21 *N*-((2*S*,3*S*,4a*R*,5*S*,10*R*,10a*R*)-2,3-Dimethoxy-2,3-dimethyl-7-phenethyl-10-(((triisopropylsilyl)oxy)methyl)-2,3,4a,5,10,10a-hexahydro-[1,4]dioxino[2,3-*d*]imidazo[1,2-*a*]pyridin-5-yl)isobutyramide (**31**)^41^

Prepared from **28** in 78% yield as described for preparation of **29** with replacement of acetic anhydride for *iso*-butyric anhydride.

*δ*_H_ (500 MHz, CDCl_3_) 7.19–7.15 (2H, m, *Ph*), 7.12–7.05 (3H, m, *Ph*), 6.86 (1H, s, H-3), 4.52 (1H, t, *J* 9.9 Hz, H-7), 4.44–4.36 (1H, br t, H-8), 4.22 (1H, dd, *J*_5*a,5*b_ 11.0, *J*_5*a,5_ 1.7 Hz, H-5^∗^a), 4.05 (1H, ddd, *J*_5,6_ 9.9, *J*_5*b,5_ 6.2, *J*_5*a,5_ 1.3 Hz, H-5), 3.88 (1H, dd, *J*_5*a,5*b_ 11.0, *J*_5*b,5_ 6.1 Hz, H-5^∗^b), 3.71 (1H, t, *J*_6,5_=*J*_6,7_ 10.1 Hz, H-6), 3.16 (3H, s, O*Me*), 3.09 (3H, s, O*Me*), 2.88 (1H, ddd, *J* 14.0, 11.2, 6.3 Hz, H-2′a), 2.83–2.67 (3H, m, H-2′b, H-2″a, H-2″b), 2.45–2.36 (1H, m, COC*H*(CH_3_)_2_), 1.21 (3H, s, *Me*), 1.15 (3H, s, *Me*), 1.09 (3H, d, *J* 6.9 Hz, COCH(C*H*_3_)_2_), 1.02 (3H, d, *J* 6.8 Hz, COCH(C*H*_3_)_2_), 1.01–0.94 (21H, m, Si(C*H*(*Me*)_2_)_3_).

*δ*_C_ (126 MHz, CDCl_3_) 177.3 (*C*OCH(*C*H_3_)_2_), 143.7 (C-8a), 142.1, 141.1, 128.3, 128.2, 125.7, 113.5 (C-3), 99.3, 99.2, 67.9 (C-7), 65.7 (C-6), 62.6 (C-5^∗^), 59.1 (C-5), 49.3 (C-8), 48.1, 47.6, 35.6 (C-2′), 35.2 (CO*C*H(CH_3_)_2_), 30.2 (C-2″), 20.0 (COCH(*C*H_3_)_2_), 19.2 (COCH(*C*H_3_)_2_), 18.0, 17.9, 17.6, 17.4, 11.9. *R_f_*=0.35; [DCM/PE 1:1]-Me_2_CO 30%, [α]_D_ +101.4 (*c* 1.0, CHCl_3_). HRMS-(TOF): MH^+^, found 644.4105. C_35_H_58_N_3_O_6_Si requires 644.4095.

#### 4.1.22 *N*-((2*S*,3*S*,4a*R*,5*S*,10*R*,10a*R*)-2,3-Dimethoxy-2,3-dimethyl-7-phenethyl-10-(((triisopropylsilyl)oxy)methyl)-2,3,4a,5,10,10a-hexahydro-[1,4]dioxino[2,3-*d*]imidazo[1,2-*a*]pyridin-5-yl)pentanamide (**32**)^41^

A solution of **28** (0.05 g, 0.084 mmol) in MeOH (1.5 mL) was stirred under slight positive pressure of H_2_ (balloon) of H_2_ in the presence of 20% Pd_2_(OH)_2_ on carbon (0.05 g) for 2 h. The reaction was filtered through a pad of Celite and concentrated in vacuum. The residue was dissolved in DCM (2 mL) and treated with valeric acid (0.027 mL, 0.25 mmol), PyBOP (0.13 g, 0.25 mmol) and DIPEA (0.5 mL) for 2 h at rt. The reaction was diluted with DCM and washed successively with 1 M HCl, water and a mixture of saturated NaHCO_3_ solution and brine. The aqueous layers were additionally extracted with DCM. The combined organic layer was dried and concentrated. The residue was purified by flash chromatography Tol/Me_2_CO 5→25% to give 0.046 g (0.076 mmol, 90%) of the title compound **32** as foam.

*δ*_H_ (500 MHz, CDCl_3_) 7.20–7.16 (2H, m, *Ph*), 7.13–7.07 (3H, m, *Ph*), 6.88 (1H, s, H-3), 4.50 (1H, t, *J*_7,6_=*J*_7,8_=10.0 Hz, H-7), 4.41–4.32 (1H, br t, H-8), 4.21 (1H, dd, *J*_5*a,5*b_ 11.0, *J*_5*a,5_ 1.8 Hz, H-5^∗^a), 4.04 (1H, ddd, *J*_5,6_ 9.7, *J*_5*b,5_ 5.8, *J*_5*a,5_ 1.5 Hz, H-5), 3.90 (1H, dd, *J*_5*a,5*b_ 11.0, *J*_5*b,5_ 5.9 Hz, H-5^∗^b), 3.72 (1H, t, *J*_6,7_=*J*_6,5_=10.0 Hz, H-6), 3.16 (3H, s, O*Me*), 3.07 (3H, s O*Me*), 2.98–2.87 (1H, m, H-2′a), 2.83–2.68 (3H, m, H-2′b, H-2″a, H-2″b), 2.26 (1H, dt, *J* 15.1, 7.7 Hz, COC*H*_2_C_2_H_4_CH_3_), 2.21–2.11 (1H, m, COC*H*_2_CH_2_CH_2_CH_3_), 1.58–1.48 (2H, m, COCH_2_C*H*_2_CH_2_CH_3_), 1.28 (2H, dq, *J* 14.7, 7.4 Hz, COCH_2_CH_2_C*H*_2_CH_3_), 1.21 (3H, s, *Me*), 1.13 (3H, s, *Me*), 1.07–0.93 (21H, m, Si(C*H*(*Me*)_2_)_3_), 0.79 (3H, t, *J* 7.4 Hz, COCH_2_CH_2_CH_2_C*H*_3_).

*δ*_C_ (126 MHz, CDCl_3_) 173.4 (*C*OCH_2_CH_2_CH_2_CH_3_), 143.6 (C-8a), 142.1, 141.4, 128.3, 128.2, 125.7, 113.4 (C-3), 99.3, 99.1, 67.9 (C-7), 65.7 (C-6), 62.6 (C-5^∗^), 59.0 (C-5), 49.4 (C-8), 48.1, 47.6, 36.4 (CO*C*H_2_CH_2_CH_2_CH_3_), 35.7 (C-2′), 30.5 (C-2″), 27.8 (COCH_2_*C*H_2_CH_2_CH_3_), 22.4 (COCH_2_CH_2_*C*H_2_CH_3_), 18.0, 17.9, 17.5, 17.4, 13.8 (COCH_2_CH_2_CH_2_*C*H_3_), 11.9. *R_f_*=0.32; Tol/Me_2_CO 20%, [α]_D_ +101.3 (*c* 0.35, CHCl_3_). HRMS-(TOF): MH^+^, found 658.4239. C_36_H_60_N_3_O_6_Si requires 658.4251.

#### 4.1.23 (*E*)-*N*-((2*S*,3*S*,4a*R*,5*S*,10*R*,10a*R*)-2,3-Dimethoxy-2,3-dimethyl-7-phenethyl-10-(((triisopropylsilyl)oxy)methyl)-2,3,4a,5,10,10a-hexahydro-[1,4]dioxino[2,3-*d*]imidazo[1,2-*a*]pyridin-5-yl)penta-2,4-dienamide (**33**)^41^

Prepared from **28** in 93% yield as described for preparation of **32** with replacement of valeric acid for 2,4-pentanedienic acid.

*δ*_H_ (500 MHz, CDCl_3_) 7.16 (2H, m, *Ph*), 7.11–7.05 (3H, m, *Ph*), 7.00 (1H, dd, *J* 15.1, 11.0 Hz, COCHC*H*CHCH_2_), 6.96 (1H, s, H-3), 6.26 (1H, dt, *J* 17.0, 10.5, 10.5 Hz, COCHCHC*H*CH_2_), 6.00 (1H, d, *J* 15.2 Hz, COC*H*CHCHCH_2_), 5.34 (1H, d, *J*_trans_ 16.9 Hz, COCHCHCHC*H*_2_), 5.23 (1H, d, *J*_cis_ 10.1 Hz, COCHCHCHC*H*_2_), 4.63–4.43 (2H, m, H-7, H-8), 4.24 (1H, d, *J*_5*a,5*b_ 11 Hz, H-5^∗^a), 4.09 (1H, dd, *J*_5,6_ 9.9, *J*_5*b,5_ 6.5 Hz, H-5), 3.92 (1H, dd, *J*_5*a,5*b_ 11.1, *J*_5*b,5_ 6.1 Hz, H-5^∗^b), 3.71 (1H, t, *J*_6,7_=*J*_6,5_=10.0 Hz, H-6), 3.15 (3H, s, O*Me*), 2.98 (3H, s, O*Me*), 2.92 (1H, m, H-2′a), 2.84–2.70 (3H, m, H-2′b, H-2″a, H-2″b), 1.20 (3H, s, *Me*), 1.06 (3H, s, *Me*), 1.05–0.91 (21H, m, Si(C*H*(*Me*)_2_)_3_).

*δ*_C_ (126 MHz, CDCl_3_) *δ* 164.2 (*C*OCHCHCHCH_2_), 152.3, 143.2 (C-8a), 142.1 (COCH*C*HCHCH_2_), 135.2 (COCHCH*C*HCH_2_), 132.9, 128.6, 127.7, 125.9 (COHCHCHCH_2_), 121.7 (COCHCHCH*C*H_2_), 118.5, 116.1 (C-3), 99.3, 99.1, 68.1 (C-7), 65.3 (C-6), 63.0 (C-5^∗^), 58.7 (C-5), 49.7 (C-8), 48.6, 47.9, 35.4 (C-2′), 35.0 (C-2″), 18.0, 17.7, 17.5, 17.4, 12.0. *R_f_*=0.3; Tol/Me_2_CO 20%. [α]_D_ +97.4 (*c* 0.85, CHCl_3_). HRMS-(TOF): MH^+^, found 654.3943. C_36_H_60_N_3_O_6_Si requires 654.3938.

#### 4.1.24 *S*-(3-(((2*S*,3*S*,4a*R*,5*S*,10*R*,10a*R*)-2,3-Dimethoxy-2,3-dimethyl-7-phenethyl-10-(((triisopropylsilyl)oxy)methyl)-2,3,4a,5,10,10a-hexahydro-[1,4]dioxino[2,3-*d*]imidazo[1,2-*a*]pyridin-5-yl)amino)-3-oxopropyl) ethanethioate (**34**)^41^

Prepared from **28** in 74% yield as described for preparation of **32** with replacement of valeric acid for *S*-acetyl-3-mercaptopropionic acid.

*δ*_H_ (500 MHz, CDCl_3_) 7.21–7.15 (2H, m, *Ph*), 7.12–7.07 (3H, m, *Ph*), 6.87 (1H, s, H-3), 4.46 (1H, t, *J* 9.9 Hz, H-7), 4.4 (1H, br t, H-8), 4.21 (1H, d, *J* 10.8 Hz, H-5^∗^a), 4.03 (1H, dd, *J* 9.8, 5.8 Hz, H-5), 3.89 (1H, dd, *J* 10.9, 5.9 Hz, H-5^∗^b), 3.72 (1H, t, *J* 9.9 Hz, H-6), 3.15 (3H, s, O*Me*), 3.09 (3H, s, O*Me*), 3.02 (2H, m, H-2′a, H-2′b), 2.92 (1H, m, H-2″a), 2.75 (3H, m, H-2″b, COC*H*_2_CH_2_SAc), 2.51 (2H, m, COCH_2_C*H*_2_SAc), 2.17 (3H, s, SCOC*H*_3_), 1.22 (3H, s, *Me*), 1.15 (3H, s, *Me*), 1.02–0.95 (21H, m, Si(C*H*(*Me*)_2_)_3_).

*δ*_C_ (126 MHz, CDCl_3_) 195.7 (S*C*OCH_3_), 170.8 (*C*OC_2_H_4_SAc), 143.2 (C-8a), 142.1, 141.7, 128.4, 128.2, 125.8, 113.6 (C-3), 99.3, 99.2, 68.0 (C-7), 65.7 (C-6), 62.7 (C-5^∗^), 59.1 (C-5), 49.6 (C-8), 48.1, 47.8, 36.1 (CO*C*H_2_*C*H_2_SAc), 35.7 (C-2′), 30.4 (C-2″), 24.7 (SCO*C*H_3_), 18.0, 17.9, 17.6, 17.4, 11.9. *R_f_*=0.35; Tol/EA 20%, [α]_D_ +101.4 (*c* 1.0, CHCl_3_). HRMS-(TOF): MH^+^, found 704.3763. C_36_H_58_N_3_O_7_SSi requires 704.3765.

#### 4.1.25 *N*-((5*R*,6*R*,7*R*,8*S*)-6,7-Dihydroxy-5-(hydroxymethyl)-2-phenethyl-5,6,7,8-tetrahydroimidazo[1,2-*a*]pyridin-8-yl)acetamide (**35**)^41^

A solution of **29** (0.046 g, 0.075 mmol) in 95% trifluoroacetic acid (2 mL) was kept for 36 h at rt. The reaction was diluted with toluene, concentrated in vacuum and co-evaporated with toluene once more. The residue was purified on Phenomenex Luna 21×100 5 μm C18(2) column, gradient 5–95% MeCN in water (0.1% NH_3_) at flow rate 25 mL/min. Appropriate fractions were pooled, concentrated to approximately 1/3 of the initial volume in vacuum and freeze dried to give 0.02 g (0.058 mmol, 77%) of the title compound **35** as amorphous solid.

*δ*_H_ (500 MHz, CDCl_3_/CD_3_OD) 7.07–7.01 (2H, m, *Ph*), 6.98–6.92 (3H, m, *Ph*), 6.65 (1H, s, H-3), 4.68 (1H, d, *J*_8,7_ 8.3 Hz, H-8), 3.86 (1H, dd, *J*_5*a,5*b_ 12.2, *J*_5*a,5_ 2.8 Hz, H-5^∗^a), 3.71 (1H, dd, *J*_5*a,5*b_ 12.2, *J*_5*b,5_ 4 Hz, H-5^∗^b), 3.63 (2H, m, H-5, H-6), 3.53 (1H, dd, *J*_7,8_ 8.5, *J*_7,6_ 8.3 Hz, H-7), 2.66 (2H, m, H-2′a, H-2′b), 2.57 (2H, m, H-2″a, H-2″b), 1.86 (3H, s, COC*H*_3_).

*δ*_C_ (126 MHz, CDCl_3_/CD_3_OD) *δ* 177.2 (*C*OCH_3_), 146.5, 144.4 (C-8a), 130.8, 128.5 (C-2), 117.2 (C-3), 78.0 (C-7), 72.8 (C-6), 64.4 (C-5), 64.2(C-5^∗^), 54.5 (C-8), 37.9 (C-2′), 34.1 (C-2″), 22.4 (CO*C*H_3_). HRMS-(TOF): MH^+^, found 346.1761. C_18_H_24_N_3_O_4_ requires 346.1767.

#### 4.1.26 *N*-((5*R*,6*R*,7*R*,8S)-6,7-Dihydroxy-5-(hydroxymethyl)-2-phenethyl-5,6,7,8-tetrahydroimidazo[1,2-*a*]pyridin-8-yl)propionamide (**36**)^41^

Prepared starting from compound **30** as described for **35** in 73% yield; amorphous solid.

*δ*_H_ (500 MHz, pyridine-*d*_5_) 8.97 (1H, d, *J*_NH,8_ 8.2 Hz, N*H*COC_2_H_5_), 7.40 (1H, s, H-3), 7.30 (3H, m, *Ph*), 7.21 (2H, m, *Ph*), 5.85 (1H, t, *J*_8,7_=*J*_NH,8_=8.5 Hz, H-8), 4.67 (1H, dd, *J*_5*a,5*b_ 11.5, *J*_5*a,5_ 1.8 Hz, H-5^∗^a), 4.59 (1H, t, *J*_6,7_=*J*_6,5_=8.6 Hz, H-6), 4.43 (2H, m, H-5^∗^b, H-7), 4.32 (1H, ddd, *J*_5,6_ 8.6, *J*_5*b,5_5.4, *J*_5*a,5_ 2.4 Hz, H-5), 3.07 (4H, m, H-2′a, H-2′b, H-2″a, H-2″b), 2.45 (2H, m, COC*H*_2_CH_3_), 1.21 (3H, t, *J*=7.5 Hz, COCH_2_C*H*_3_).

*δ*_C_ (126 MHz, pyridine-*d*_5_) 176.8 (*C*OCH_2_CH_3_), 146.5, 144.9, 144.4 (C-8a), 130.8, 130.7, 128.1 (C-2), 116.4 (C-3), 77.5 (C-7), 72.3 (C-6), 64.7 (C-5), 64.0 (C-5^∗^), 53.7 (C-8), 38.3 (C-2′), 33.3 (C-2″), 31.7 (CO*C*H_2_CH_3_), 12 (COCH_2_*C*H_3_). HRMS-(TOF): MH^+^, found 360.1922. C_19_H_26_N_3_O_4_ requires 360.1923.

#### 4.1.27 *N*-((5*R*,6*R*,7*R*,8*S*)-6,7-Dihydroxy-5-(hydroxymethyl)-2-phenethyl-5,6,7,8-tetrahydroimidazo[1,2-*a*]pyridin-8-yl)isobutyramide (**37**)^41^

Prepared starting from compound **31** as described for **35** in 70% yield; amorphous solid.

*δ*_H_ (500 MHz, pyridine-*d*_5_) 8.81 (1H, br d, *J*_NH,8_ N*H*CO), 7.28 (1H, br s, H-3), 7.17–7.2 (4H, m, *Ph*), 7.07–7.11 (1H, m, *Ph*), 5.73 (1H, t, *J*_8,7_=*J*_NH,8_=8.4 Hz, H-8), 4.55 (1H, dd, *J*_5*a,5*b_ 11.7, 2.6 Hz, H-5^∗^a), 4.47 (1H, t, *J*_7,6_=*J*_7,8_=8.6 Hz, H-7), 4.3 (2H, m, H-5^∗^b, H-6), 4.2 (1H, m, H-5), 2.95 (4H, m, H-2′a, H-2′b, H-2″a, H-2″b), 2.66 (1H, quint, COC*H*(CH_3_)_2_), 1.19 (3H, d, *J* 6.8 Hz, COCH(C*H*_3_)_2_), 1.18 (3H, d, *J* 6.8 Hz, COCH(C*H*_3_)_2_).

*δ*_C_ (126 MHz, pyridine-*d*_5_) 180.1 (*C*OCH(CH_3_)_2_), 146.4, 144.9, 144.4 (C-8a), 130.8, 130.7, 128.1 (C-2), 116.5 (C-3), 77.6 (C-7), 72.3 (C-6), 64.8 (C-5), 64.1 (C-5^∗^), 53.7 (C-8), 38.3 (C-2′), 37.7 (C-2″), 33.3 (CO*C*H(CH_3_)_2_), 22.1 (COCH(*C*H_3_)_2_), 21.9 (COCH(*C*H_3_)_2_). HRMS-(TOF): MH^+^, found 374.2075. C_20_H_28_N_3_O_4_ requires 374.2080.

#### 4.1.28 *N*-((5*R*,6*R*,7*R*,8*S*)-6,7-Dihydroxy-5-(hydroxymethyl)-2-phenethyl-5,6,7,8-tetrahydroimidazo[1,2-*a*]pyridin-8-yl)pentanamide (**38**)^41^

Prepared starting from compound **32** as described for **35** in 68% yield; amorphous solid.

*δ*_H_ (500 MHz, DMSO) 7.98 (1H, d, *J*_NH,8_ 9.0 Hz, N*H*CO), 7.32–7.22 (4H, m, *Ph*), 7.21–7.15 (1H, m, *Ph*), 7.01 (1H, s, H-3), 5.42 (1H, d, *J* 4.8 Hz, O*H*-6), 5.24 (1H, d, *J* 4.8 Hz, O*H*-7), 4.98 (1H, t, *J* 7.6 Hz, O*H*-5^∗^), 4.75 (1H, t, *J*_8,7_=*J*_8,NH_=8.8 Hz, H-8), 4.00 (1H, ddd, *J*_5*a,5*b_ 11.3, 4.4, 1.4 Hz, H-5^∗^a), 3.75–3.68 (1H, m, H-5^∗^b), 3.69–3.61 (2H, m, H-6, H-5), 3.62–3.53 (1H, m, H-7), 2.91–2.78 (2H, m, H-2′a, H-2′b), 2.68 (2H, t, *J* 8.3 Hz, H-2″a, H-2″b), 2.15 (2H, t, *J* 7.4 Hz, COC*H*_2_CH_2_CH_2_CH_3_), 1.59–1.47 (2H, m, COCH_2_C*H*_2_CH_2_CH_3_), 1.44–1.30 (2H, m, COCH_2_CH_2_C*H*_2_CH_3_), 0.89 (3H, t, *J* 7.3 Hz, COC_3_H_6_C*H*_3_).

*δ*_C_ (126 MHz, DMSO d_6_) 173.4 (NH*C*O), 144.5, 142.1 (C-8a), 141.6, 129.0, 126.7, 113.9 (C-3), 73.3 (C-6), 69.4 (C-5/C-7), 61.9 (C-5/C-7), 61.1 (C-5^∗^), 50.2 (C-8), 36.0 (CO*C*H_2_CH_2_CH_2_CH_3_, C-2′), 31.2 (C-2″), 28.3 (COCH_2_*C*H_2_CH_2_CH_3_), 22.6 (COCH_2_CH_2_*C*H_2_CH_3_), 14.3 (COCH_2_CH_2_CH_2_*C*H_3_). HRMS-(TOF): MH^+^, found 388.2232. C_21_H_30_N_3_O_4_ requires 388.2236.

#### 4.1.29 (*E*)-*N*-((5*R*,6*R*,7*R*,8*S*)-6,7-Dihydroxy-5-(hydroxymethyl)-2-phenethyl-5,6,7,8-tetrahydroimidazo[1,2-*a*]pyridin-8-yl)penta-2,4-dienamide (**39**)^41^

Prepared staring from compound **33** as described for **35** in 48% yield; amorphous solid.

*δ*_H_ (500 MHz, DMSO) 8.39 (1H, d, *J*_NH,8_ 8.7 Hz, N*H*CO), 7.31–7.23 (4H, m, *Ph*), 7.22–7.14 (1H, m, *Ph*), 7.09 (1H, dd, *J* 15.2, 11.1 Hz, COCHC*H*CHCH_2_), 7.06 (1H, s, H-3), 6.54 (1H, dt, *J* 16.9, 10.5 Hz, COCHCHC*H*CH_2_), 6.15 (1H, d, *J* 15.2 Hz, COC*H*CHCHCH_2_), 5.63 (1H, d, *J*_trans_ 16.7 Hz, COCHCHCHC*H*_2_), 5.44 (1H, d, *J*_cis_ 11.4 Hz, COCHCHCHC*H*_2_), 5.5 (1H, br s, O*H*-6), 5.38 (1H, br s, O*H*-7), 5.03 (1H, br s, O*H*-5^∗^), 4.86 (1H, t, *J*_8,7_=*J*_8,NH_=8.9 Hz, H-8), 4.01 (1H, dd, *J*_5*a,5*b_ 10.9, 2.8 Hz, H-5^∗^a), 3.79–3.64 (3H, m, H-5, H-5^∗^b, H-6), 3.63–3.56 (1H, m, H-7), 2.93–2.73 (2H, m, H-2′a, H-2′b), 2.68 (2H, t, *J* 8.1 Hz, H-2″a, H-2″b).

*δ*_C_ (126 MHz, DMSO d_6_) 164.7 (*C*OCHCHCHCH_2_), 158.8, 152.7, 143.1 (C-8a), 141.9 (COCH*C*HCHCH_2_), 139.3, 135.2 (COCHCH*C*HCH_2_), 128.2, 128.0, 126.6, 125.7 (CO*C*HCHCHCH_2_), 123.8 (COCHCHCH*C*H_2_), 113.6(C-3), 72.8 (C-7), 68.5 (C-6), 61.0 (C-5^∗^), 60.2 (C-5), 49.5 (C-8), 38.8 (C-2′), 35.2 (C-2″). HRMS-(TOF): MH^+^, found 384.1919. C_21_H_26_N_3_O_4_ requires 384.1923.

#### 4.1.30 *S*-(3-(((5*R*,6*R*,7*R*,8*S*)-6,7-Dihydroxy-5-(hydroxymethyl)-2-phenethyl-5,6,7,8-tetrahydroimidazo[1,2-*a*]pyridin-8-yl)amino)-3-oxopropyl) ethanethioate (**40**)^41^

Prepared starting from compound **34** as described for **35** in 75% yield; amorphous solid.

*δ*_H_ (500 MHz, pyridine-*d*_5_) 9.31 (1H, d, *J*_NH,8_ 8.4 Hz, N*H*CO), 7.40 (1H, s, H-3), 7.33–7.27 (2H, m, *Ph*), 7.24–7.17 (3H, m, *Ph*), 5.87 (1H, t, *J*_8,7_=*J*_8,NH_=8.6 Hz, H-8), 4.67 (1H, dd, *J*_5*a,5*b_ 11.4, *J*_5*a,5_ 1.6 Hz, H-5^∗^a), 4.60 (1H, t, *J*_6,7_=*J*_6,5_=8.6 Hz, H-6), 4.44 (2H, m, H-5^∗^b, H-7), 4.32 (1H, ddd, *J*_5,6_ 8.6, *J*_5*b,5_ 4.7, *J*_5*a,5_ 1.6 Hz, H-5), 3.52–3.36 (2H, m), 3.17–2.97 (4H, m), 2.96–2.82 (2H, m), 2.16 (3H, s, SCOC*H*_3_).

*δ*_C_ (126 MHz, pyridine-*d*_5_) 197.4 (S*C*OCH_3_), 173.8 (*C*ONH), 146.3, 144.9, 144.4 (C-8a), 130.8, 130.7, 128.1, 116.5 (C-3), 77.2 (C-7), 72.3 (C-6), 64.8 (C-5), 64.0 (C-5^∗^), 53.8 (C-8), 38.4, 38.2, 33.3, 32.3 (SCO*C*H_3_), 27.3. HRMS-(TOF): MH^+^, found 434.1756. C_21_H_28_N_3_O_5_S requires 434.1750.

#### 4.1.31 *N*-((5*R*,6*R*,7*R*,8*S*)-6,7-Dihydroxy-5-(hydroxymethyl)-2-phenethyl-5,6,7,8-tetrahydroimidazo[1,2-*a*]pyridin-8-yl)-3-mercaptopropanamide (**41**)^41^

To a solution of **40** (0.042 g, 0.1 mmol) and dithiothreitol (0.015 g) in DMF (1.5 mL) stock 25% solution of MeONa in MeOH (0.03 mL) was added at rt. The reaction was stirred for 2 h and quenched with AcOH (0.05 mL). The reaction was concentrated in vacuum and the residue was purified on the C18(2) column as described above to give 0.023 g (0.056 mmol, 56%) of the title compound **41** as amorphous solid.

*δ*_H_ (500 MHz, MeOD) 7.29 (2H, m, *Ph*), 7.24 (1H, s, H-3), 7.23–7.15 (3H, m, *Ph*), 4.90 (1H, d, *J*_8,7_ 7.6 Hz, H-8), 4.15 (1H, dd, *J*_5*a,5*b_ 12.1, 2.6 Hz, H-5^∗^a), 4.10–4.05 (1H, m, H-5), 4.02 (1H, dd, *J*_7,6_ 8.5, *J*_7,8_ 7.8 Hz, H-7), 3.99 (1H, dd, *J*_5*a,5*b_ 12.5, 5.2 Hz, H-5^∗^b), 3.92 (1H, dd, *J*_6,7_ 8.6, *J*_6,5_ 7.7 Hz, H-6), 2.98–2.88 (4H, m), 2.84–2.74 (2H, m), 2.70–2.60 (2H, m).

*δ*_C_ (126 MHz, MeOD) 174.5 (NH*C*O), 145.3, 144.9, 144.4 (C-8a), 130.8, 128.7, 128.5, 126.7, 116.5 (C-3), 71.0 (C-7), 68.1 (C-6), 62.9 (C-5), 60.1 (C-5^∗^), 49.2 (C-8), 39.8, 34.8, 27.6, 20.0. HRMS-(TOF): MH^+^, found 392.1641. C_19_H_26_N_3_O_4_S requires 392.1644.

## Mol files

The following ZIP file contains the MOL files of the most important compounds referred to in this article.

MOL files ZIP file containing the MOL files of the most important compounds in this article.
